# Hepatitis C virus enhances Rubicon expression, leading to autophagy inhibition and intracellular innate immune activation

**DOI:** 10.1038/s41598-020-72294-y

**Published:** 2020-09-17

**Authors:** Yuto Shiode, Hayato Hikita, Satoshi Tanaka, Kumiko Shirai, Akira Doi, Sadatsugu Sakane, Yugo Kai, Tasuku Nakabori, Ryoko Yamada, Takahiro Kodama, Ryohei Narumi, Ryotaro Sakamori, Hidetoshi Eguchi, Takeshi Tomonaga, Tomohide Tatsumi, Tetsuo Takehara

**Affiliations:** 1 Department of Gastroenterology and Hepatology, Osaka University Graduate School of Medicine, 2-2 Yamadaoka, Suita, Osaka 565-0871 Japan; 2 Laboratory of Proteome Research/Proteome for Drug Discovery, National Institute of Biomedical Innovation, Health and Nutrition, 7-6-8, Saito-Asagi, Ibaraki, Osaka 567-0085 Japan; 3 Department of Gastroenterological Surgery, Osaka University Graduate School of Medicine, Suita, Osaka 565-0871 Japan

**Keywords:** Gastroenterology, Hepatology

## Abstract

Autophagy, a degradation system, works to maintain cellular homeostasis. However, as the impact of Hepatitis C virus (HCV) infection on hepatocyte autophagy and its effect on HCV replication remain unclear, we examined them. HCV infection suppressed late-stage autophagy and increased Rubicon. siRNA-mediated knockdown of Rubicon promoted autophagy in HCV-infected cells. In Huh-7 cells harbouring the HCV replicon, Rubicon knockdown downregulated the expression of type 1 interferon (IFN)-related genes and upregulated HCV replication. Rubicon overexpression or administration of bafilomycin A1 or chloroquine, an inhibitor of late-stage autophagy, suppressed autophagy and activated the type 1 IFN pathway. On the other hand, Atg7 knockout suppressed early-stage autophagy and did not activate the type 1 IFN pathway. In livers of humanized liver chimeric mice, HCV infection increased Rubicon and enhanced type 1 IFN signalling. Elimination of HCV in the mice reduced the increase in Rubicon due to HCV infection. The expression levels of Rubicon and IFN-stimulated genes in chronic hepatitis C patients were higher than those in non-B, non-C hepatitis patients. HCV infection increased Rubicon and suppressed hepatocyte autophagy, leading to activation of the intracellular immune response. Rubicon induction is involved in HCV replication via activation of the intracellular immune response.

## Introduction

Hepatitis C virus (HCV) is a positive-stranded RNA virus of the Flaviviridae family. Approximately 100 million people are affected with HCV worldwide^1^. Chronic infection with HCV leads to liver cirrhosis and hepatocellular carcinoma^2^. Although most HCV-infected patients treated with direct-acting antivirals (DAAs) can achieve virologic cure, the development of HCV resistance to DAAs remains a problematic issue^3–6^.

Autophagy is a highly conserved cellular degradation process in virtually all eukaryotic cells that plays a pivotal role in removing the cells’ own damaged organelles and long-lived proteins to maintain cellular homeostasis^7,8^. Autophagosomes have been reported to accumulate in HCV-infected hepatocytes^9^. Shrivastava et al. reported that HCV infection activates mTOR signalling and autophagy via upregulation of Beclin1^10^. It was also reported that HCV infection induces complete autophagy, which promotes HCV replication^11^. In contrast, Mori et al. reported that HCV infection impairs autophagic flux^12^. Whether autophagy is enhanced or suppressed by HCV remains controversial.

In the present study, we examined whether autophagy is promoted by HCV infection and its effect on HCV replication in HCV-infected cells and livers. Here, we demonstrated that HCV infection suppressed autophagy by increasing Rubicon, a Beclin1‐interacting negative regulator of autophagosome‐lysosome fusion. Rubicon activated the intracellular immune response and suppressed HCV replication. The present study reveals the impact of the HCV-induced increase in Ruction on the intracellular immune system.

## Results

### HCV infection suppressed hepatocyte autophagy by upregulating Rubicon expression

**Figure 1:**
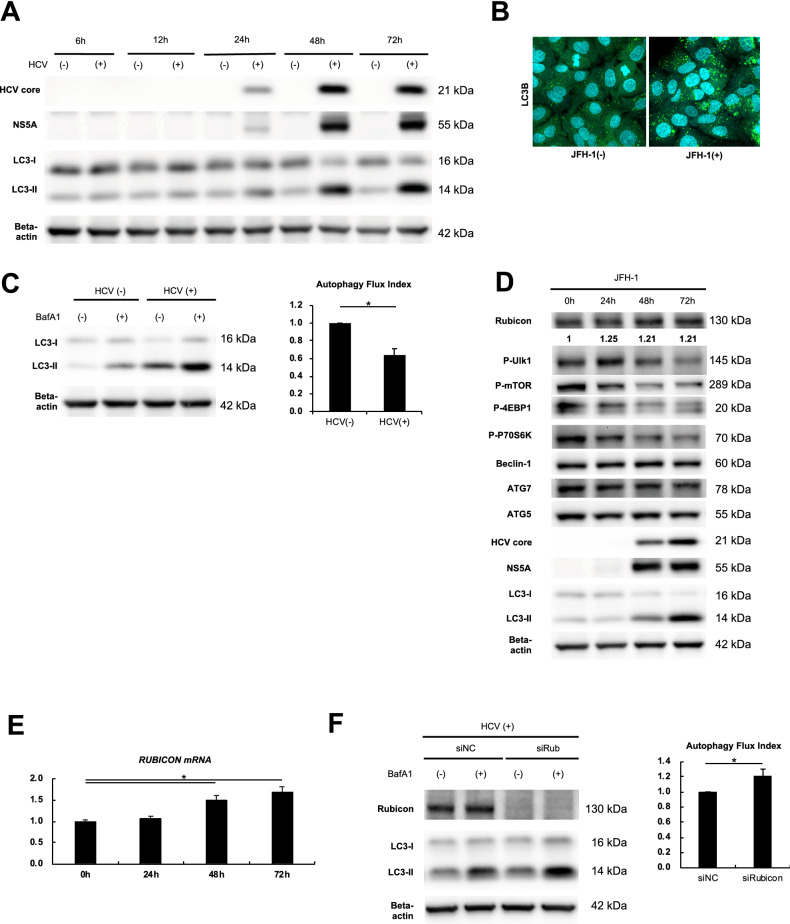
HCV infection suppressed hepatocyte autophagy through upregulation of Rubicon expression. (**A–E**) Huh7.5.1 cells were infected with HCV (JFH-1) and then harvested at the indicated times. Western blot analysis of the HCV core, HCV NS5A and LC3 (A). Representative confocal microscopy images of Huh7.5.1 cells and JFH-1-infected Huh7.5.1 cells at 72 h after inoculation (**B**). Immunofluorescent staining was performed with DAPI (blue) and a monoclonal antibody against LC3 (green). LC3 turnover assay to determine the autophagic flux index using bafilomycin, a late-stage inhibitor of autophagy, at 72 h after inoculation (**C**); n = 3 per group. Western blot analysis of HCV core, HCV NS5A and autophagy-related proteins (**D**). The mRNA expression level of Rubicon (**E**). (**F**) Huh7.5.1 cells were transfected with siRNA against Rubicon (siRub) or negative control siRNA (siNC) for 72 h and infected with JFH-1 for 48 h. LC3 turnover assay to determine the autophagic flux index using bafilomycin, a late-stage inhibitor of autophagy (n = 3 per each). *p < 0.05.

The expression levels of LC3-II, a constituent of membranes that correlates with the number of autophagosomes^13^, were increased in a time-dependent manner from 24 h after inoculation with HCV (JFH-1), as shown by western blotting (Fig. 1A). At 3 days after inoculation, most of the cells expressed NS5A, an HCV nonstructural protein, and the number of LC3 puncta, which correlates with the number of autophagosomes, had increased (Sup. Figure 1A, Fig. 1B). At that time, protein expression levels of P62, which is specifically degraded by autophagy, increased. However, this increase could not be directly interpreted as a result of an autophagy supression because P62 mRNA expression levels were also increased at that time (Sup. Figure 1C). Therefore, we examined autophagy flux to evaluate autophagy progression. The autophagy flux index determined using bafilomycin A1, a negative regulator of autophagosome-lysosome fusion, was decreased by HCV infection (Fig. 1C), suggesting that HCV infection suppressed autophagy at a late stage in hepatocytes. To investigate the molecular mechanism of autophagy suppression by HCV infection, the expression levels of autophagy-related proteins were evaluated by western blotting (Fig. 1D). The expression levels of mTOR pathway proteins (phospho-ULK1, phospho-mTOR, phospho-4EBP1, phospho-P70S6K), which negatively regulate autophagy initiation, decreased in a time-dependent manner after HCV infection. The expression levels of major autophagy pathway proteins involved in autophagosome formation (Beclin-1, Atg7, Atg5) were unaltered by HCV infection. In contrast, the expression level of Rubicon, which negatively regulates autophagosome-lysosome fusion, increased in a time-dependent manner after HCV infection (Fig. 1D). The transcription of Rubicon was increased by HCV infection in a time-dependent manner after HCV infection (Fig. 1E). siRNA-mediated knockdown of Rubicon reversed autophagy suppression in HCV-infected cells (Fig. 1F), suggesting that increased expression of Rubicon is involved in autophagy suppression by HCV infection. Similar to observations in JFH-1-infected cells, the gene expression level of Rubicon was increased and autophagy was suppressed at a late stage in Huh7 cells transfected with the HCV subgenome compared with untransfected Huh7 cells (Sup. Figure 1D). Huh7.5.1 cells can be efficiently infected with JFH-1, but a report demonstrated that Huh7.5.1 cells have a mutation in their retinoic acid inducible gene-I (RIG-I) gene that facilitates detection of double-stranded RNA in the cytoplasm and Interferon regulatory factor-3- and NF-kB-mediated activation of IFN^14^. Therefore, the following studies were performed using Huh7 cells harbouring the HCV subgenomic replicon.

### Rubicon activates intracellular immunity

**Figure 2:**
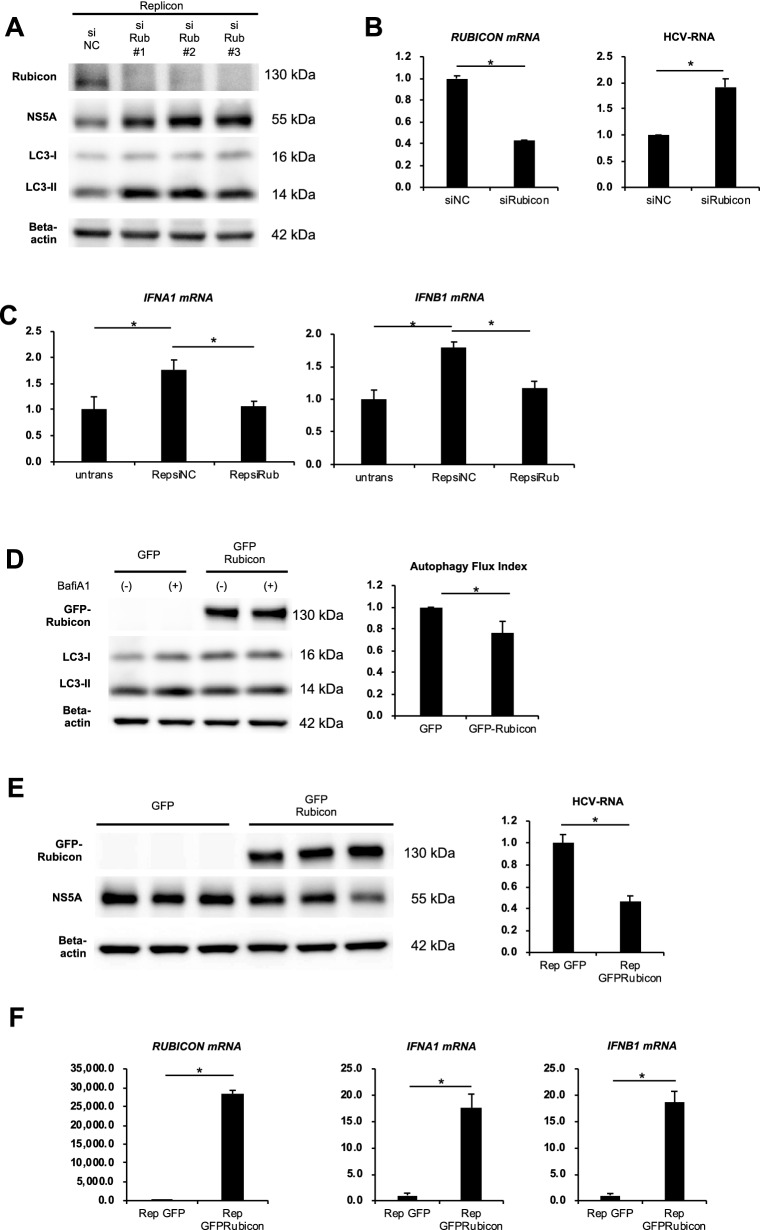
Rubicon activated innate immunity. (**A–C**) HCV replicon-harbouring cells were transfected with three different siRNAs against Rubicon (siRub #1, #2 and #3) for 72 h. Western blot analysis of HCV NS5A, LC3 and Rubicon (**A**). The mRNA expression levels of Rubicon and HCV RNA levels (**B**). The mRNA expression levels of type 1 interferon-related genes (**C**). (**D–F**) HCV replicon-harbouring cells were transfected with GFP plasmid or GFP-Rubicon plasmid for 72 h. LC3 turnover assay to determine autophagic flux index using bafilomycin (**D**). mRNA expression levels of Rubicon and type 1 interferon-related genes (**E**). HCV RNA levels in cells and western blot analysis of HCV NS5A (F). n = 3 per each experiment. *p < 0.05.

The protein expression levels of NS5A and HCV RNA in HCV subgenome-transfected cells were increased by Rubicon silencing (Fig. 2A,B). Expression levels of the type I IFN-related genes IFNA1 and IFNB1 were higher in HCV subgenome-transfected cells than in untransfected cells and decreased by Rubicon knockdown (Fig. 2C). Forced expression of Rubicon suppressed autophagy (Fig. 2D), increased type I IFN-related gene expression (Fig. 2F) and decreased expression levels of NS5A and HCV RNA (Fig. 2E,F) in HCV replicon-harbouring cells. Forced expression of Rubicon rescued the Rubicon knockdown-induced increase in HCV RNA and decreases in IFNA1 and IFNB1 levels (Sup. Figure 2).

### Autophagy suppression at a late stage activated intracellular immunity

**Figure 3:**
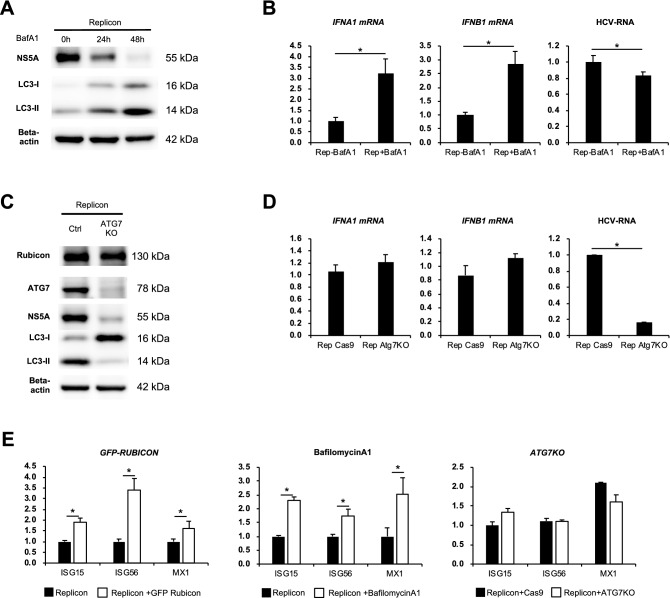
Late-stage autophagy inhibition activated innate immunity. (**A**, **B**) HCV replicon-harbouring cells were treated with 125 nM bafilomycin A1 for the indicated durations. Western blot analysis of HCV NS5A and LC3 (**A**). The mRNA expression levels of type 1 interferon-related genes and HCV RNA levels in cells (B). (**C, D**) HCV replicon-harbouring cells were transfected with Crispr/Cas9 plasmids against Atg7 or a negative control plasmid (Cas9). Western blot analysis of ATG7, HCV NS5A and LC3 (**C**). Western blot analysis of HCV NS5A and LC3 (**C**). The mRNA expression levels of type 1 interferon-related genes and HCV RNA levels in cells (**D**). (**E**) The mRNA expression levels of interferon-stimulated genes (ISGs) in HCV replicon-harbouring cells transfected with GFP plasmid or GFP-Rubicon plasmid for 72 h (left panel), treated with or without 125 nM bafilomycin A1 for 24 h (middle panel), and transfected with Crispr/Cas9 plasmid against Atg7 (siAtg7) or a negative control plasmid (Cas9) (right panel). *p < 0.05.

We investigated the effect of autophagy inhibition on intracellular immunity and HCV replication. Bafilomycin A1, a negative regulator of autophagy at a late stage, increased the gene expression levels of IFNA1 and IFNB1 and decreased the expression levels of NS5A and HCV RNA in HCV replicon-harbouring cells (Fig. 3A,B). Similar results were obtained using chloroquine, another chemical inhibitor of late-stage autophagy (Sup. Figure 3A, Sup. Figure 3B). In contrast, Crispr/Cas9-mediated knockout of Atg7, a gene involved in the early stages of autophagy, did not change the expression levels of IFNA1 and IFNB1 (Fig. 3C,D).

Next, we evaluated the expression levels of IFN-stimulated genes (ISGs), which are downstream of the type I IFN signalling pathway, under several conditions that inhibit autophagy. HCV subgenome-transfected cells were transfected with GFP-Rubicon plasmid, subjected to Atg7 knockout or treated with bafilomycin A1. RT-PCR analysis revealed that the expression levels of ISG15, ISG56 and Mx1 were markedly increased by GFP-Rubicon overexpression or bafilomycin A1 treatment but unchanged by Atg7 knockout (Fig. 3E). The expression levels of ISG15 and ISG56 were increased by Rubicon silencing (Sup. Figure 3C). Forced expression of Rubicon rescued the Rubicon knockdown-induced decreases in ISG15 and ISG56 levels (Sup. Figure 2). Overexpression of ISG15, ISG56 and MX1 suppressed the HCV RNA levels in HCV subgenome-transfected cells (Sup. Figure 3D).

### HCV infection increased Rubicon in the livers of chimeric mice, which was reversed by DAA treatment

**Figure 4:**
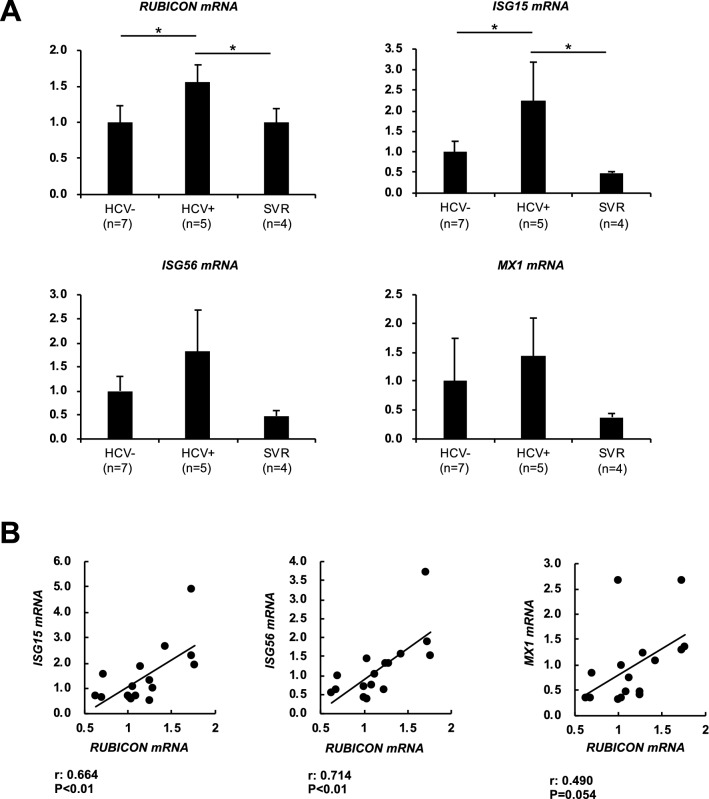
HCV infection increased Rubicon gene expression in the livers of human hepatocyte chimeric mice. Human hepatocyte chimeric mice were infected with HCV (genotype 1b) patient serum for 8 weeks. HCV-infected chimeric mice were treated and achieved sustained viral response (SVR). (**A**) The mRNA expression levels of Rubicon, Atg7 and type 1 interferon-related genes in the livers of control uninfected chimeric mice (n = 5), HCV-infected chimeric mice (n = 5) and SVR mice (n = 4). (**B**) Correlation diagram between the Rubicon gene and the ISG15, ISG56 and Mx1 genes. *p < 0.05.

To examine the reproducibility of these results in not only hepatoma cells but also human normal hepatocytes, we examined Rubicon and ISG expression levels in the livers of human liver chimeric mice upon HCV infection. The mRNA levels of Rubicon in the livers of HCV-infected chimeric mice were higher than those of uninfected chimeric mice and decreased after HCV elimination with anti-HCV treatment (Fig. 4A). Expression of ISG-related proteins in the livers of HCV-infected chimeric mice was higher than that of uninfected chimeric mice or HCV-eliminated chimeric mice (Fig. 4A). Rubicon gene expression levels in the livers of HCV-infected mice were positively correlated with expression levels of the ISG15, ISG56, and Mx1 genes (Fig. 4B).

To examine human-specific protein expression levels in the livers of HCV-infected chimeric mice and uninfected mice, liver lysates from uninfected chimeric mice (n = 5) and HCV-infected mice (n = 4) were subjected to proteome analysis. A total of 3,620 human proteins were quantified. Among them, the expression of 36 proteins was increased, while that of 9 proteins was decreased in HCV-infected livers compared to uninfected livers using a cut-off value of *p* < 0.05 (Sup. Table). These 45 proteins were significantly related to IFN-related pathways, as shown by gene ontology analysis (Table 1).

**Table 1:** List of pathways significant enriched in the proteins listed in Sup. Table 1 determined by GO analysis.

Term	Count	%	PValue	Fold Enrichment
GO: 0060337 ~ type I interferon signalling pathway	12	26.66667	1.95E−16	40.19139
GO: 0051607 ~ defence response to virus	10	22.22222	7.53E−11	23.56902
GO: 0060333 ~ interferon-gamma-mediated signalling pathway	8	17.77778	3.41E−09	28.28283
GO: 0006955 ~ immune response	7	15.55556	4.13E−06	14.84848

### Patients with chronic hepatitis C showed increased expression levels of the Rubicon gene and ISGs

**Figure 5:**
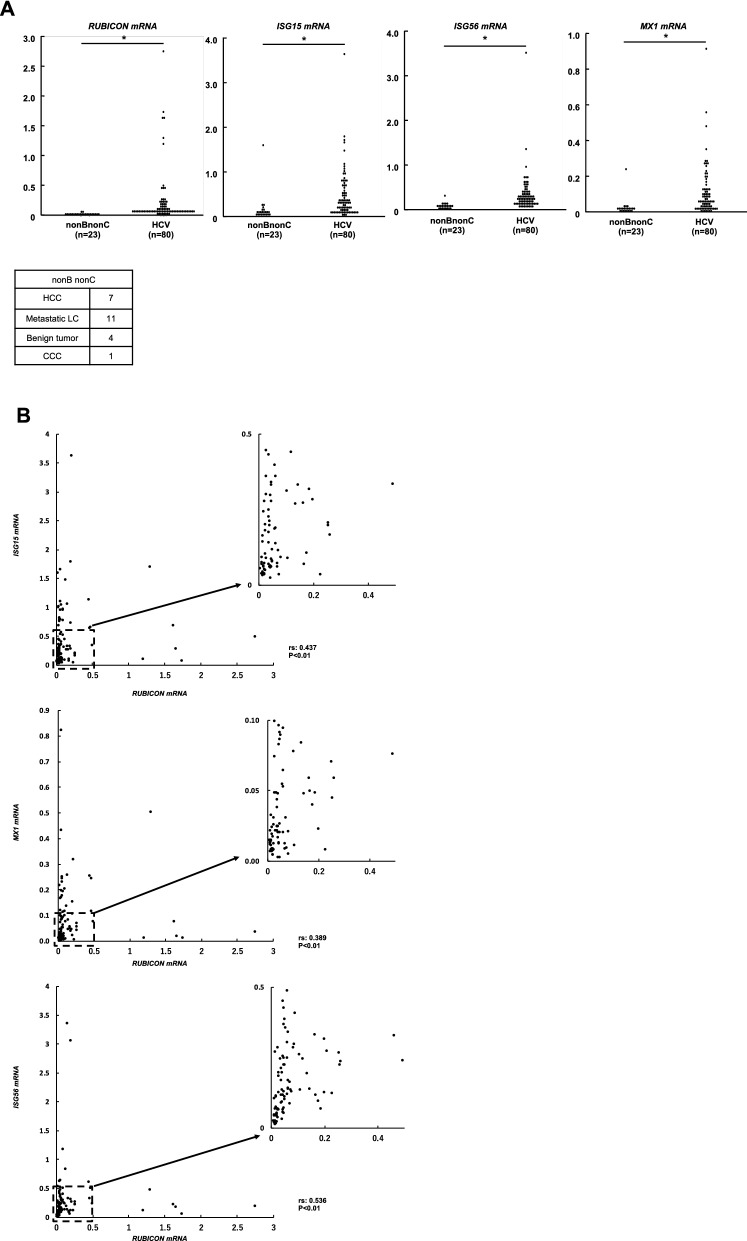
Upregulation of Rubicon in human liver samples from patients with chronic hepatitis C. Resected non-cancerous lesions from the livers of non-B non-C patients (n = 23), including 7 HCC patients, 11 metastatic liver cancer patients, 4 benign tumour patients and 1 CCC patient, and liver biopsy samples from patients with chronic hepatitis C (n = 80) were analysed. (**A**) The mRNA expression levels of Rubicon and interferon-stimulated genes. (**B**) Correlation between Rubicon gene expression levels and type 1 interferon-related gene expression levels. rs: Spearman's rank correlation coefficient. *p < 0.05.

The expression levels of autophagy- and immunity-related genes were evaluated using non-cancerous liver biopsy specimens obtained from chronic hepatitis C patients and non-B, non-C patients. In patients with chronic hepatitis C, the gene expression levels of Rubicon, ISG15, Mx1, and ISG56 were higher than those in non-B, non-C patients (Fig. 5A). In addition, when the correlation between expression levels of the Rubicon gene and each studied ISG was individually examined, ISG15 and ISG56 were correlated with Rubicon, while the correlation of Mx1 with Rubicon was weak (Fig. 5B).

## Discussion

In the present study, we clarified that hepatocyte autophagy is suppressed by HCV infection though upregulation of Rubicon. Furthermore, suppression of late-stage autophagy activates IFN signalling, which suppresses HCV replication. This study revealed that Rubicon-induced IFN signalling activation contributes to HCV replication in HCV-infected hepatocytes.

Rubicon was discovered as a protein that negatively regulates the fusion of autophagosomes and lysosomes. Although the mechanism of the Rubicon homologue in *C. elegans* has been reported^15^, the regulatory mechanism of Rubicon expression in mammals has not been clarified. There have also been several reports on Rubicon and gastrointestinal diseases. In non-alcoholic fatty liver disease (NAFLD) patients, Rubicon in the hepatocytes increases, which causes liver steatosis and hepatocyte apoptosis induction^1^. In hepatocytes, HBV increases Rubicon, which suppresses IRF3 phosphorylation, leading to type I IFN suppression via inhibition of IRF3 import to the nucleus^16^. Since Rubicon negatively regulates the fusion of autophagosomes and lysosomes, Rubicon knockdown in non-infected cells decreases the LC3-II level (Sup. Figure 4A), while Rubicon overexpression enhances the LC3-II level (Sup. Figure 4B). However, in HCV infected cells, Rubicon knockdown or Rubicon overexpression did not induce a clear change in the LC3-II level, although autophagy flux was increased or decreased, as shown in Sup. Figure 4C or Fig. 2D, respectively. The levels of LC3-II in HCV-infected cells or HCV subgenome-transfected cells with bafilomycin A1 were significantly higher than those in uninfected cells or untransfected cells with bafilomycin A1, respectively (Fig. 1A, Sup. Figure 1B), indicating that HCV itself increases the LC3-II induction. In a Rubicon knockdown experiment, while Rubicon knockdown decreased the LC3-II levels, the Rubicon knockdown-induced HCV elevation served to increase the LC3-II levels. This effect of the Rubicon knockdown-induced HCV elevation may explain why Rubicon knockdown did not induce a clear decrease in LC3-II levels (Figs. 1F, 2A), although it increased autophagy flux, as shown in Sup. Figure 4C. Similarly, the Rubicon overexpression-induced HCV suppression may explain why Rubicon overexpression did not induce a clear increase in LC3-II levels (Fig. 2D), although it decreased autophagy flux, as shown in Fig. 2D.

In the present study, forced expression of Rubicon or administration of bafilomycin A1 or chloroquine activated the intracellular type I IFN pathway. In contrast, knockout of Atg7 did not activate the intracellular type I IFN pathway. These results suggested that inhibition of hepatocellular autophagy at a late stage, but not an early stage, activated the intracellular type I IFN pathway. We could not clarify the underlying mechanisms by which inhibition of hepatocellular autophagy at a late stage activates type 1 IFN. In the present study, while Rubicon overexpression induced ISG15, ISG56 and MX1 (Fig. 3E), Rubicon knockdown reduced the expression levels of ISG15 and ISG56 but not MX1 (Sup. Figure 2E). ISGs are regulated not only by autophagy but also by HCV itself17. The Rubicon siRNA-induced HCV elevation served to upregulate the ISG expression levels, which may explain why Rubicon siRNA did not reduce the Mx1 expression levels.

We showed that inhibition of the fusion of autophagosomes and lysosomes, such as that mediated by forced Rubicon expression or the administration of bafilomycin A1, elevated the gene expression of type I IFN, ISG15, ISG56, and Mx1 and inhibited HCV replication. Various ISGs have been reported to suppress HCV replication. Overexpression of PKR, Mx1, IRF-9, GBP-1, IFI-6–16, IFI-27, 25OAS or IRF-1 in HCV replicon-harbouring cells suppressed HCV replication^18^. Forcible expression of ISG15 in HCV replicon-harbouring cells^19^ or ISG56 in JFH-1-infected cells^20^ suppressed HCV. In our experiments, forced expression of ISG15, ISG56 and MX1 suppressed HCV replication (Sup. Figure 3D). On the other hand, under Atg7-knockout conditions, which inhibit autophagosome formation, HCV replication was inhibited, but the production of type I IFN, ISG15, ISG56, and Mx1 was not changed. HCV replication is said to occur in a membranous web formed by lipid droplets and single, double, and multiple membrane-like structures created by remodelling of the ER^21^. The double membrane-like structure is a major component of the membranous web and thought to be formed by NS5A early in infection. The double membrane-like structure contains NS3, NS5A, viral RNA and LC3-II and is thought to be related to autophagosomes^22^. Knockout of Atg7 inhibited membranous web formation^23^ and thus suppressed HCV replication but did not appear to involve intracellular immune activation.

Wang et al. have reported that the suppression of Rubicon using siRNA decreased the HCV RNA level, while overexpression of Rubicon increased the HCV RNA level, mainly using as an experimental system Huh7.5 cells infected with the JFH-1 variant. Unlike these results, we demonstrated that the suppression of Rubicon using siRNA increased the HCV RNA level, while overexpression of Rubicon decreased the HCV RNA level, using Huh7 cell-derived replicons. Our study focused on how the alteration of hepatocyte autophagy by Rubicon drove intracellular immunity, and we used replicons derived from Huh7 cells because Huh7.5 cells have been reported to have a mutated RIG-I pathway^14^. The difference in the activation of the RIG-1 pathway between Huh7 and Huh7.5 cells may have led to these discrepancies.

Chronic hepatitis C more frequently accompanies fatty liver complications than chronic hepatitis B^24,25^. In the present study, we clarified that HCV infection inhibits hepatocyte autophagy. In contrast, HBV infection was reported to enhance hepatocyte autophagy^26^. Hepatocyte autophagy is involved in lipid droplet metabolism^27^. In the livers of mice fed a high-fat diet (HFD), Rubicon expression was upregulated, leading to autophagy inhibition and resulting in an increase in lipid accumulation^28^. Hepatocyte-specific Rubicon knockdown ameliorated HFD-induced lipid accumulation in the livers of the mice^28^. Taken together, the data indicate that the increase in Rubicon in HCV-infected hepatocytes may contribute to lipid accumulation in the livers of chronic hepatitis C patients via autophagy inhibition.

In conclusion, HCV infection enhances expression of the Rubicon protein and suppresses autophagic flux in hepatocytes. The suppression of autophagy in hepatocytes enhances intracellular immunity and suppresses HCV replication. In addition, upregulation of the Rubicon gene is involved in upregulation of the Rubicon protein by HCV infection, which was also confirmed in chimeric mice and human samples. Upregulation of the Rubicon gene at the time of HCV infection was confirmed to be reduced when HCV was eliminated by treatment in vivo.

## Materials and methods

### Cells and viruses

The Huh7 human hepatoma cell line was obtained from the JCRB Cell Bank, and Huh7.5.1 cells were obtained from the National Institute of Infectious Diseases; the cells were cultured at 37 °C in Dulbecco’s modified Eagle’s medium (DMEM; Nacalai Tesque, Japan) containing 10% heat-inactivated foetal calf serum (FCS). HCV strain JFH-1 and Huh7 cells harbouring the subgenome of HCV strain Con1 were obtained from the National Institute of Infectious Diseases and cultured in DMEM supplemented with 10% heat-inactivated FCS and 1 mg/mL G418 (Nacalai Tesque)^29^. All cell lines were tested negative for mycoplasma contamination.

Transfection of siRNA (5 nM) into cells was performed using Lipofectamine RNAiMAX Transfection Reagent (Thermo Fisher Scientific, Waltham, MA, USA) for 48–72 h according to the manufacturer's instructions. The set of siRNAs shown below was purchased from Thermo Fisher Scientific (Waltham, MA, USA): the control was silencer negative control #1 siRNA (AM4611, named siNC); RUBICON (279,044, 279,045 and 279,046, named siRub#1, #2 and #3, respectively). For forced expression experiments, cells were transfected with previously constructed GFP or GFP-Rubicon plasmid vector^30^ and a GFP-ISG15, GFP-ISG56 or GFP-Mx1 plasmid (OriGene Technologies, Inc., Beijing, China) for 48 h.

### HCV infection of human hepatocyte chimeric mice

The creation of chimeric mice based on NOG mice that express thymidine kinase (TK-NOG mice) has already been reported^31^. Based on the concentration of human albumin in the sera of the chimeric mice, the human hepatocyte formation rate in the chimeric mice was measured, and mice with a formation rate of 45% or more were used in the experiment. The serum human albumin concentration was measured by ELISA using the Human Albumin ELISA Quantitation Set (Bethyl Laboratories, Inc., TX). Chimera mice were intravenously injected with 100 µl of HCV patient (genotype 1b, DAA-naïve, 6.8 log IU/ml) serum. After inoculation, sera were collected from the mice every 4 weeks, and the amount of HCV RNA in 100-fold-diluted serum (with a lower measurement range of 3.2 log IU/ml serum) was measured using the COBAS TaqMan HCV test (Roche Diagnostics, Basel, Switzerland). After confirming sustained viremia, HCV-infected mice were sacrificed 12–16 weeks after HCV inoculation. As HCV-eliminated mice, we used previously reported HCV-treated mice that had achieved sustained viral response (SVR) for 3 weeks after the end of the treatment with simeprevir plus GS-558093 (a nucleotide analogue inhibitor of NS5B polymerase), ledipasvir plus GS-558093 or simeprevir and interferon (IFN)^32^. All mice were bred in an environment free of specific pathogens and given humane care with the approval of the Animal Care and Use Committee of Osaka University Medical School. The Serum from HCV patients used to infect chimeric mice were collected after informed consent was obtained with the approval of the Osaka University Hospital Ethics Committee (No. 29-011). All experiments were performed in accordance with relevant guidelines and regulations.

### Proteome analysis

The livers of human hepatocyte chimeric mice were homogenized in lysis buffer (50 mM NH_4_HCO_3_, 12 mM sodium deoxycholate, 12 mM sodium N-lauroylsarcosinate, protease inhibitor and phosphatase inhibitor) and incubated at 95 °C for 5 min. Liver proteins in the lysates were enzymatically digested on the basis of phase transfer surfactant protocol^33^. After reduction (10 mM TCEP, 30 min, 37 °C) and alkylation (20 mM iodoacetamide, 30 min, 37 °C in dark) of the liver proteins and quenching the reaction by 21 mM L-cysteine, they were digested with 1:50 (w/w) of trypsin (proteomics grade; Roche, Mannheim, Germany) and lysyl endopeptidase (Wako Pure Chemical Industries) at 37 °C overnight. After digestion, the detergents were removed by acidifying with 1% trifluoroacetic acid and centrifuge. The supernatants were desalted with a pipette-tip column of C18 resin as previously reported^34^. The digests were labelled with the reagents for isobaric-peptides-labelling (TMT-10 plex, Thermo Fisher Scientific) according to each manufacturer’s protocol. The TMT-labelled sample mixture was separated into 14 fractions using a HPLC system (Prominence UFLC, Shimadzu, Japan) with C18 column (2.0 × 150 mm, L-column3, Chemicals Evaluation and Research Institute, Japan). The fractions were analysed by an Orbitrap mass spectrometer (positive mode, scan range of 350–1,800 m/z, 70 K FWHM resolution, Q-Exactive plus, Thermo Fisher Scientific), which was equipped with UltiMate 3,000 nano-LC systems (Thermo Scientific). The analytical column was a capillary column (0.075 × 300 mm) packed with 1.9-μm resin (ReproSil-Pur C18-AQ, Dr. Maisch GmbH, Germany). Mobile phases were RP-A (0.1% formic acid and 2% acetonitrile) and RP-B (0.1% formic acid and 90% acetonitrile). The LC gradient was ramped up from 5 to 35% RP-B for 145 min at the flow rate of 280 nL/min. The acquired data was searched using MaxQuant software (version 1.6.3.3)^35^ against UniProt human and mouse protein database (a precursor mass tolerance of 7 ppm, a fragment ion mass tolerance of 0.01 Da, fixed modification of carbamidomethylation at cysteine, variable modification of oxidation at methionine, deamination of asparagine and glutamine). Identified peptides were accepted with a false discovery rate of < 1%. Of the identified peptides with human protein sequences, those with sequences shared with mouse proteins were excluded. The remaining peptides were used to quantify human proteins.

### Clinical samples

Non-tumourous liver samples were obtained from 83 patients who underwent liver biopsy to diagnose hepatitis or hepatocellular carcinoma and 23 patients who underwent hepatectomy and stored at -80 °C until use. Eighty patients were positive for HCV infection, and twenty-three patients were negative for hepatitis B virus or HCV infection. The patients had a daily alcohol intake of 20 g or less and had not received chemotherapy or radiation therapy within 3 months of the liver biopsy. These samples were obtained with approval of the Institutional Research Board of Osaka University Hospital (No. 15267). Informed consent was also obtained in writing from each patient. All experiments were performed in accordance with relevant guidelines and regulations.

### Western blotting

The western blotting procedure has been previously described^28^. Protein eluting solution of cells were prepared using lysis buffer with protease inhibitor and phosphatase inhibitor. We used the following antibodies for immunodetection: anti-SQSTM1/p62 (#5114), anti-LC3B (#3868), anti-Beclin1 (#3495), anti-Atg5 (#8540), anti-Atg7 (#2631), anti-phospho-mTOR (Ser2448) (#5536), anti-phospho-4EBP1 (Ser65) (#1443), anti-phospho-P70S6K (Thr389) (#9234), anti-Rubicon (#8465), and anti-phospho-ULK1 (Ser757) (#6888), which were obtained from Cell Signaling Technology (Beverly, MA); anti-β-actin (A5316), which was obtained from Sigma-Aldrich (St. Louis, MO); and anti-NS5A (ab13833) and anti-HCV core (ab2740), which were obtained from Abcam. Rabbit anti-NS5A antibody was a kind gift from the National Institute of Infectious Diseases. The expression level of each protein was corrected with the expression level of each β-actin using ImageJ software.

### Real-time reverse transcription-PCR (RT-PCR)

Total RNA extraction from cell lines and human liver tissues was performed using the RNeasy Mini kit (QIAGEN, Venlo, Netherlands). Then, 1 μg of total RNA was reverse transcribed using the ReverTra Ace qPCR RT Kit (Toyobo, Tokyo, Japan) to synthesize complementary DNA. The TaqMan gene expression assay was used to analyze the following mRNA expression: human KIAA0226 or RUBICON (Hs00943561_g1), human SQSTM1/p62 (Hs01061917_g1), human IFNA1 (Hs00855471_g1), human IFNB1 (Hs01077958_s1), human ISG15 (Hs01921425_s1), human ISG56 (Hs03027069_s1), human MX1 (Hs00895608_m1), human ATG7 (Hs00893766_m1) and human β-ACTIN (Hs01060665_g1). The following HCV RNA primers were designed: JFH-1 (FW-primer: GTCCGATGGAGAAGAAGGTCATC, RV-primer: ACGGGAAGTCCATGTAGAATGTC, MGB probe: CGGAGACGGCTGCAT) and Con1 (FW-primer: GGCTGCATCATCACTAGCCTC, RV-primer: CAGGAAAGATTGTGTTGCGGT, MGB probe: ACAGGAACCAGGTCGAG). Human gene expression levels were normalized to β-actin expression levels.

### Autophagic flux assay

Two hours before harvest, bafilomycin A1 (BioViotica, Dransfeld, Germany), a chemical autophagy inhibitor, was administrated to cells at a concentration of 125 nM, and evaluation was performed by western blotting. The autophagic flux index was calculated as the ratio of the expression level of LC3-II in the presence of bafilomycin A1 to the expression level of LC3-II in the absence of bafilomycin A1. The expression level of LC3-II was corrected by the expression level of each β-actin. The autophagy flux index for the control group in each experiment was corrected to 1.

### Immunofluorescence and confocal microscopy

Cells were washed with PBS 3 times and fixed with 4% paraformaldehyde for 20 min at room temperature. After 3 washes with PBS, the cells were quenched with 15 mM Gly-PBS at room temperature for 5 min. Cells were permeabilized with 0.1% Triton X-100 for 5 min at room temperature. After 1 wash with PBS, the cells were blocked with 0.2% gelatine-PBS for 30 min at room temperature. Cells were incubated with specific primary antibodies (LC3B Ab at a dilution of 1:1,000, NS5A Ab at a dilution of 1:1,000). After being washed with PBS 2 times, cells were incubated with secondary antibody at a dilution of 1:1,000 and Hoechst dye at a dilution of 1:5,000. The images were analysed with a CQ-1 laser scanning confocal microscope (Yokogawa Electric Corporation, Tokyo, Japan).

### Statistical analysis

The data are presented as the mean plus or minus the standard deviation. All data were subjected to appropriate statistical tests and met the test assumptions, and the levels of variance between compared groups were similar.

Comparisons between two groups were performed by unpaired two-tailed T-test for normally distributed variables and Mann–Whitney’s U-test for non-normally distributed variables. Comparison between 3 groups was carried out by the Tukey method. Differences for which *p* < 0.05 were considered to be statistically significant.

## Supplementary information

Supplementary file1

Supplementary file2

Supplementary file3

Supplementary file4

## Abbreviations

KD: Knockdown
RT-PCR: Reverse transcription-PCR
HCV: Hepatitis C virus
DAA: Direct-acting antivirals
DMEM: Dulbecco’s modified Eagle’s medium
FCS: Fetal calf serum
TK-NOG mice: NOG mice that express thymidine kinase
SVR: Sustained viral response
IFN: Interferon
ISGs: IFN-stimulated genes
HFD: High-fat diet
