# Supplementary material for: Hepatitis C virus enhances Rubicon expression, leading to autophagy inhibition and intracellular innate immune activation
Source: Sci Rep. 2020 Sep 17;10:15290. doi: 10.1038/s41598-020-72294-y (PMC7498609; doi:10.1038/s41598-020-72294-y)
Supplement: Supplementary file 1 — Supplementary file1 [file 41598_2020_72294_MOESM1_ESM.pdf]

Raw Data of Gel blots

Raw Data of figures below are shown in order. All blots were captured with full resolution mode of FUSION Solo S (Vilber Lourmat, Collégien, France) .

Fig. S1

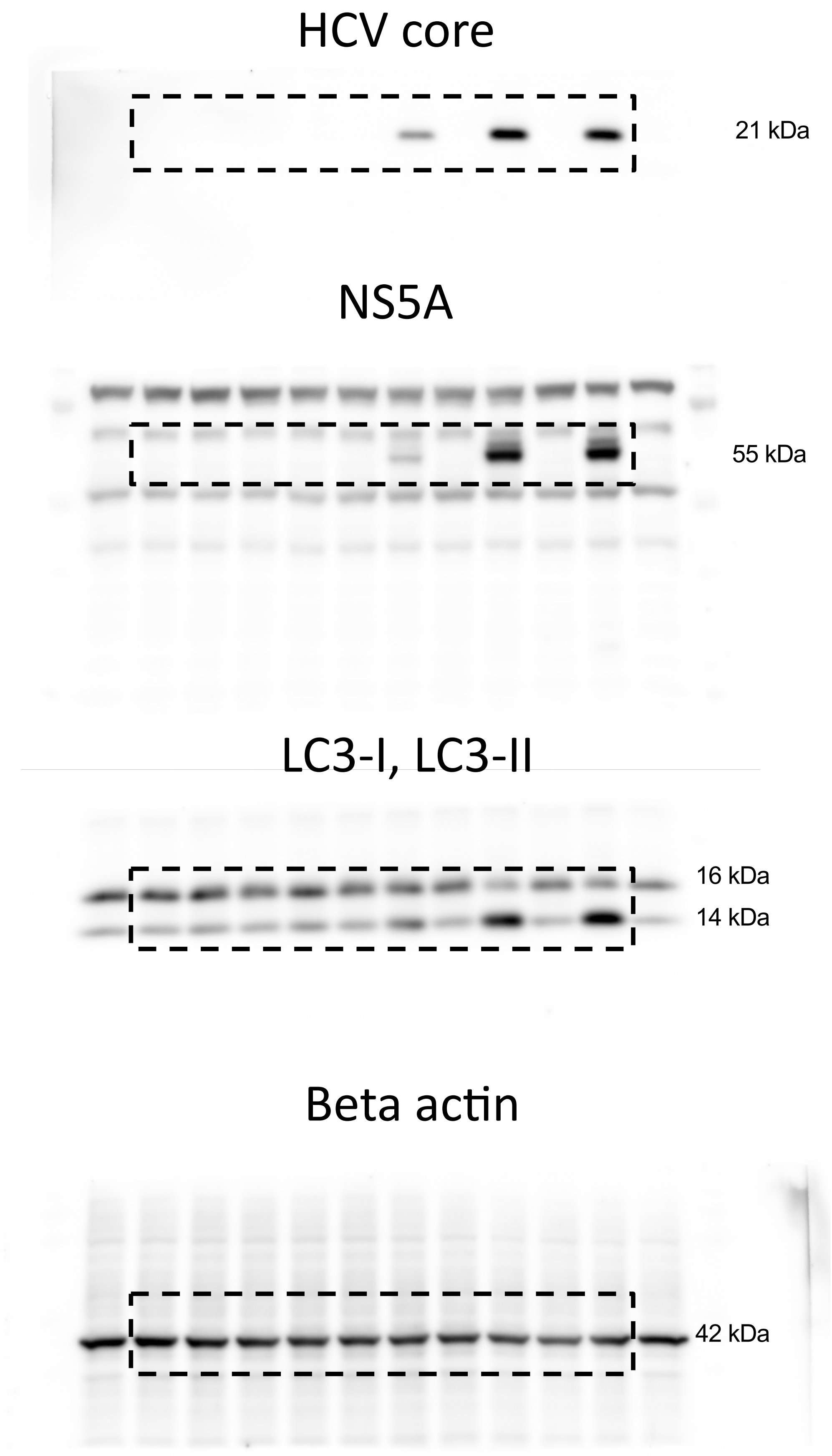

Fig. S1 Full-length images of the immunoblots in Fig. 1A. Black dot line boxes indicate the cropped images used in Fig. 1A.

Fig. S2

LC3-I, LC3-II

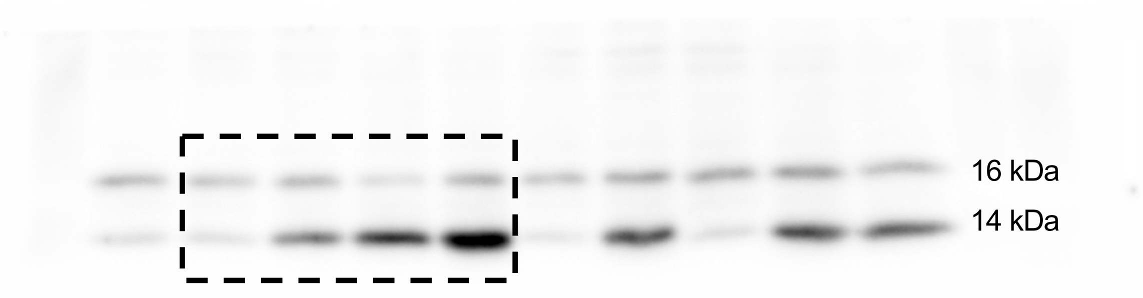

Beta actin

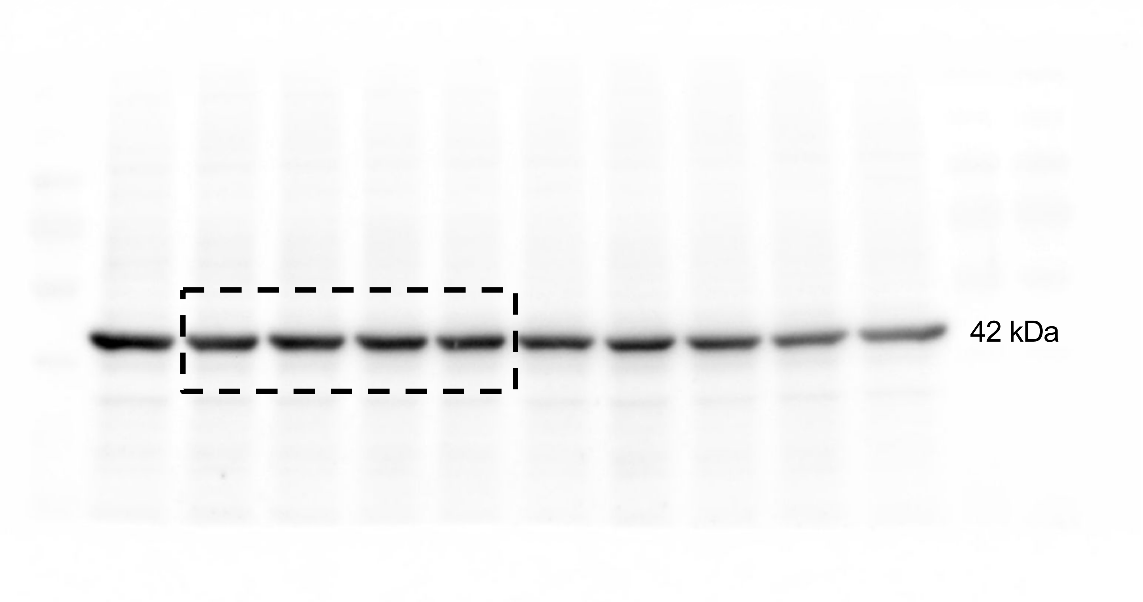

Fig. S2. Full-length images of the immunoblots in Fig. 1C. Black dot line boxes indicate the cropped images used in Fig. 1C.

Fig. S3

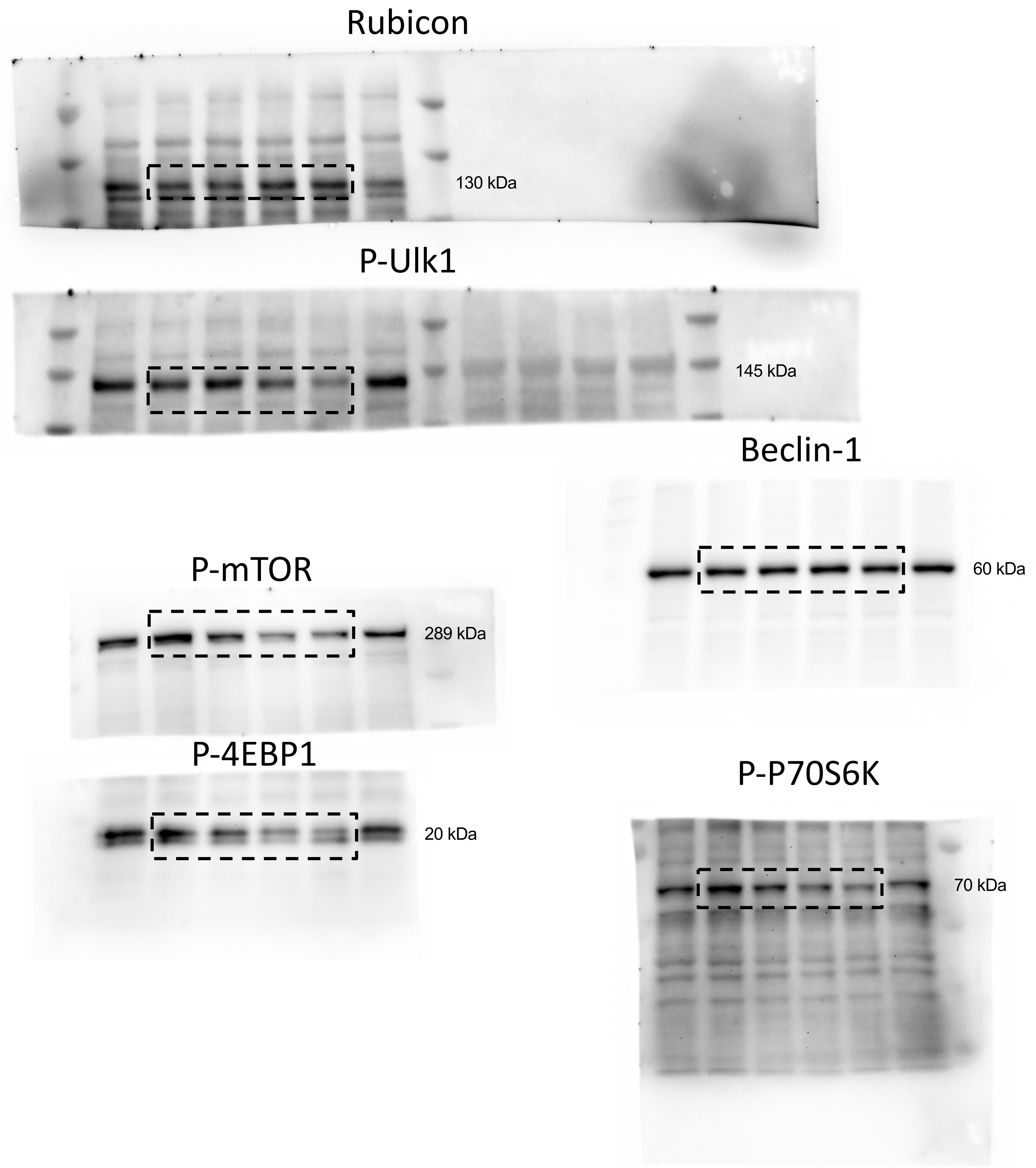

Fig. S3. Full-length images of the immunoblots in Fig. 1D. Black dot line boxes indicate the cropped images used in Fig. 1D.

Fig. S4

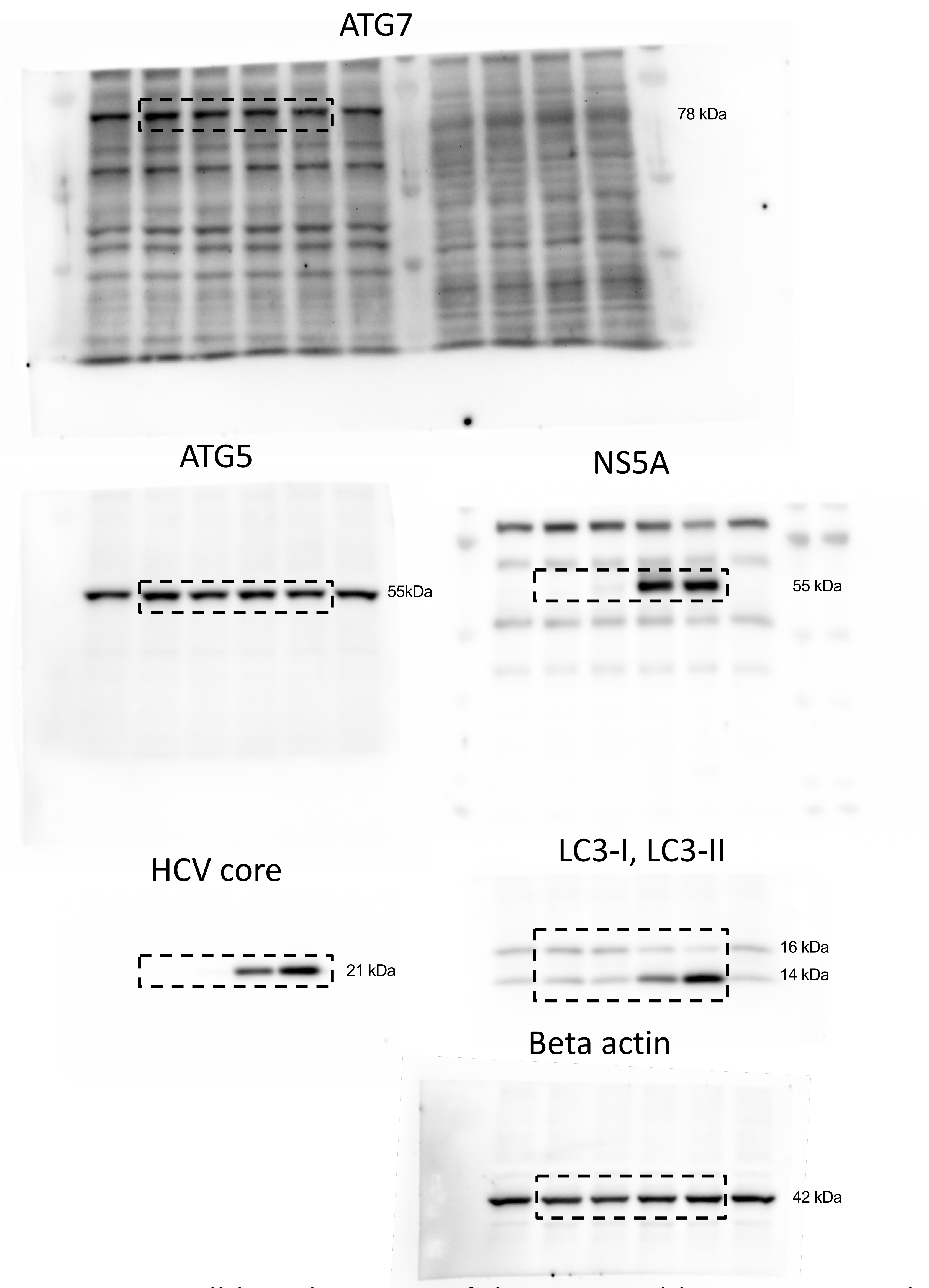

Fig. S4. Full-length images of the immunoblots in Fig. 1D. Black dot line boxes indicate the cropped images used in Fig. 1D.

Fig. S5

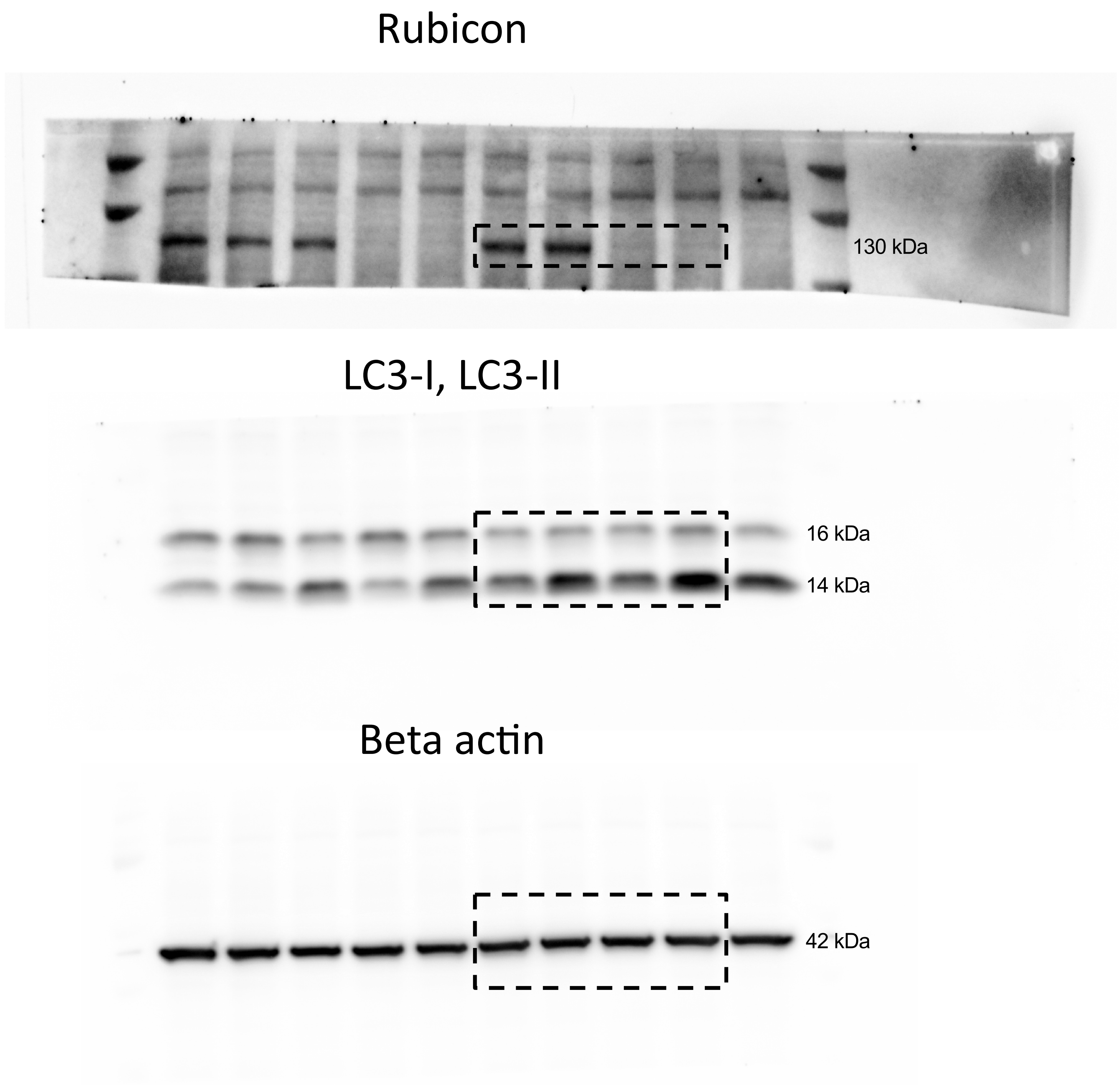

Fig. S5. Full-length images of the immunoblots in Fig. 1F. Black dot line boxes indicate the cropped images used in Fig. 1F.

Fig. S6

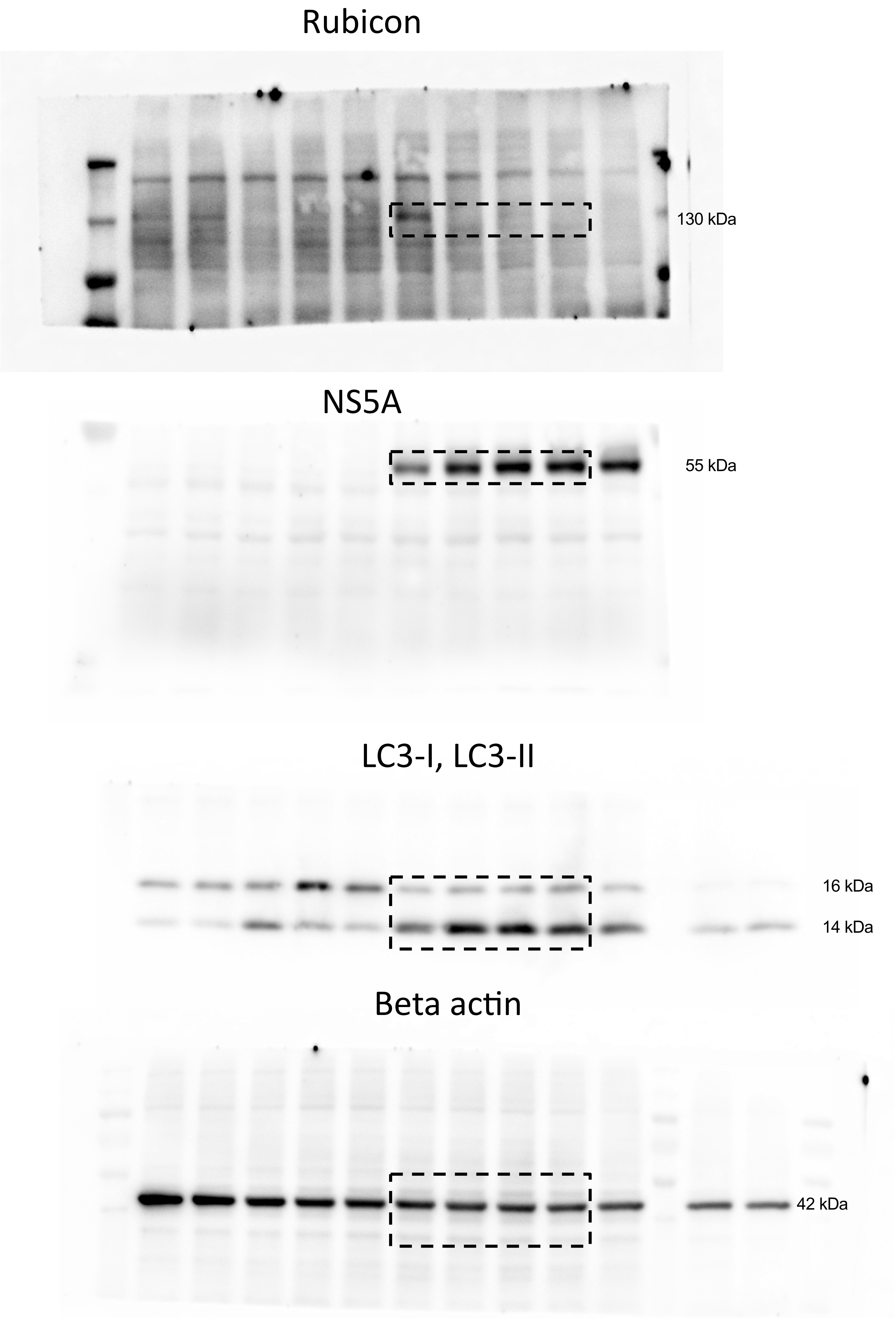

Fig. S6. Full-length images of the immunoblots in Fig. 2A. Black dot line boxes indicate the cropped images used in Fig. 2A.

Fig. S7

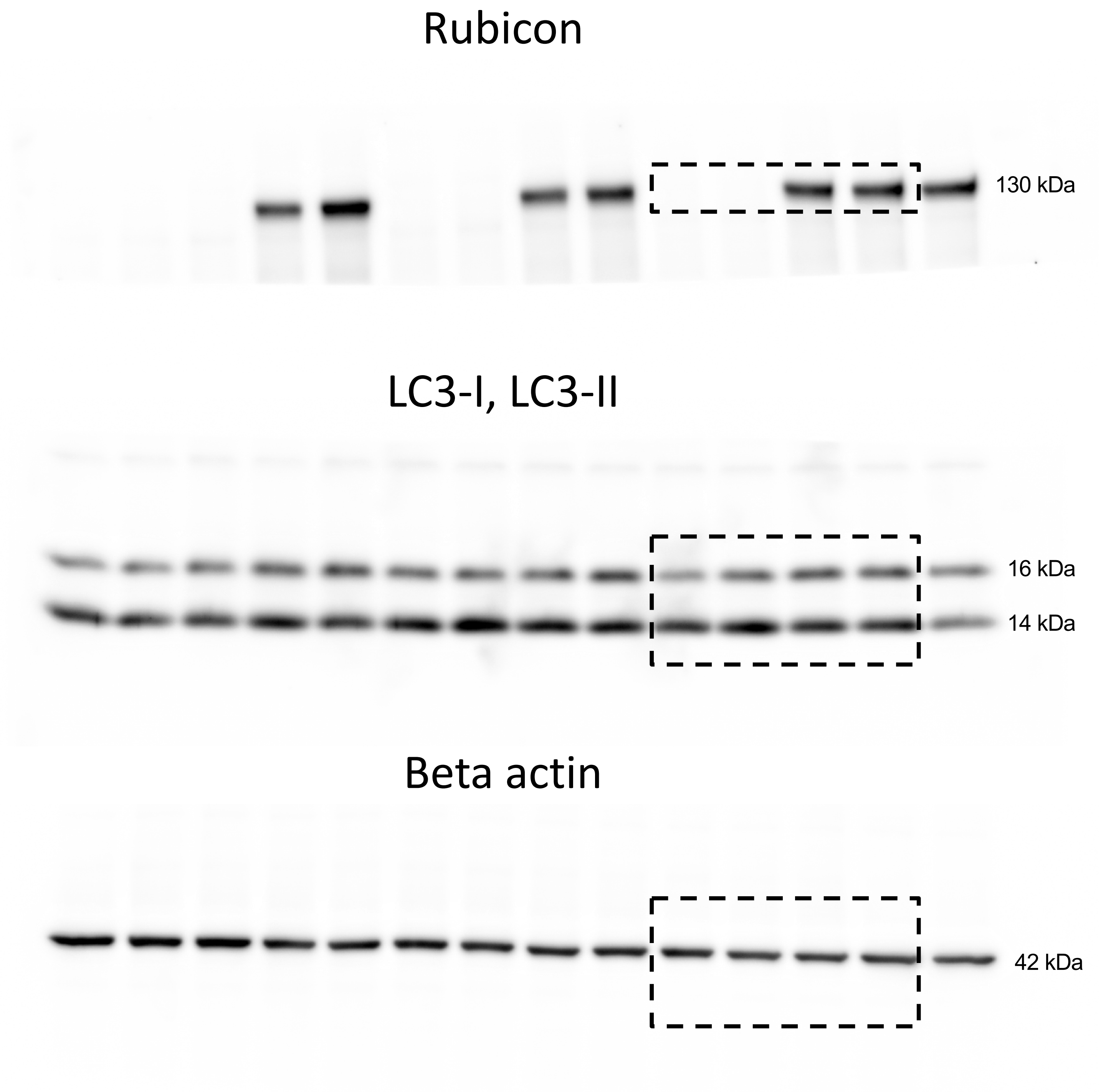

Fig. S7. Full-length images of the immunoblots in Fig. 2D. Black dot line boxes indicate the cropped images used in Fig. 2D.

Fig. S8

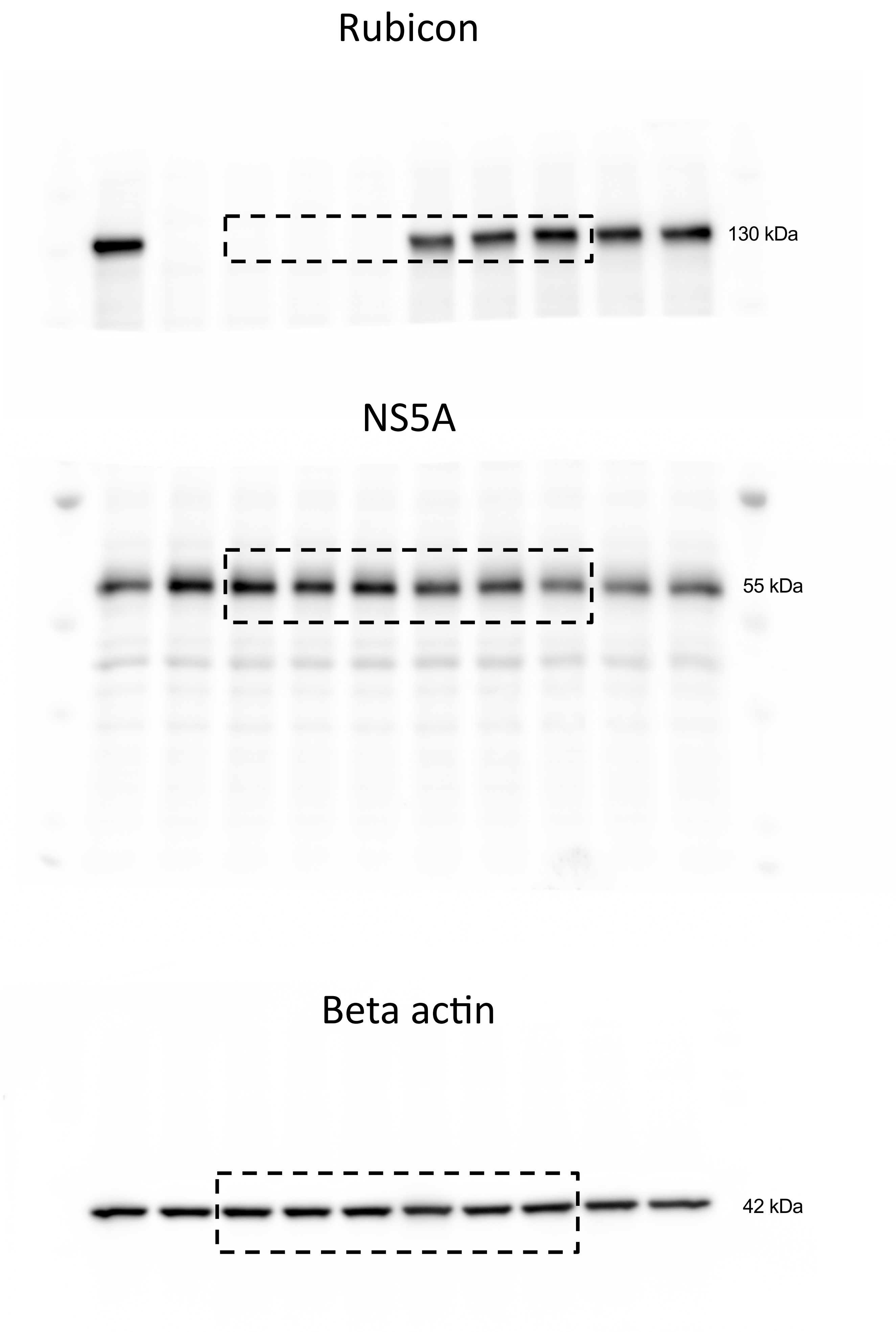

Fig. S8. Full-length images of the immunoblots in Fig. 2E. Black dot line boxes indicate the cropped images used in Fig. 2E.

Fig. S9

NS5A

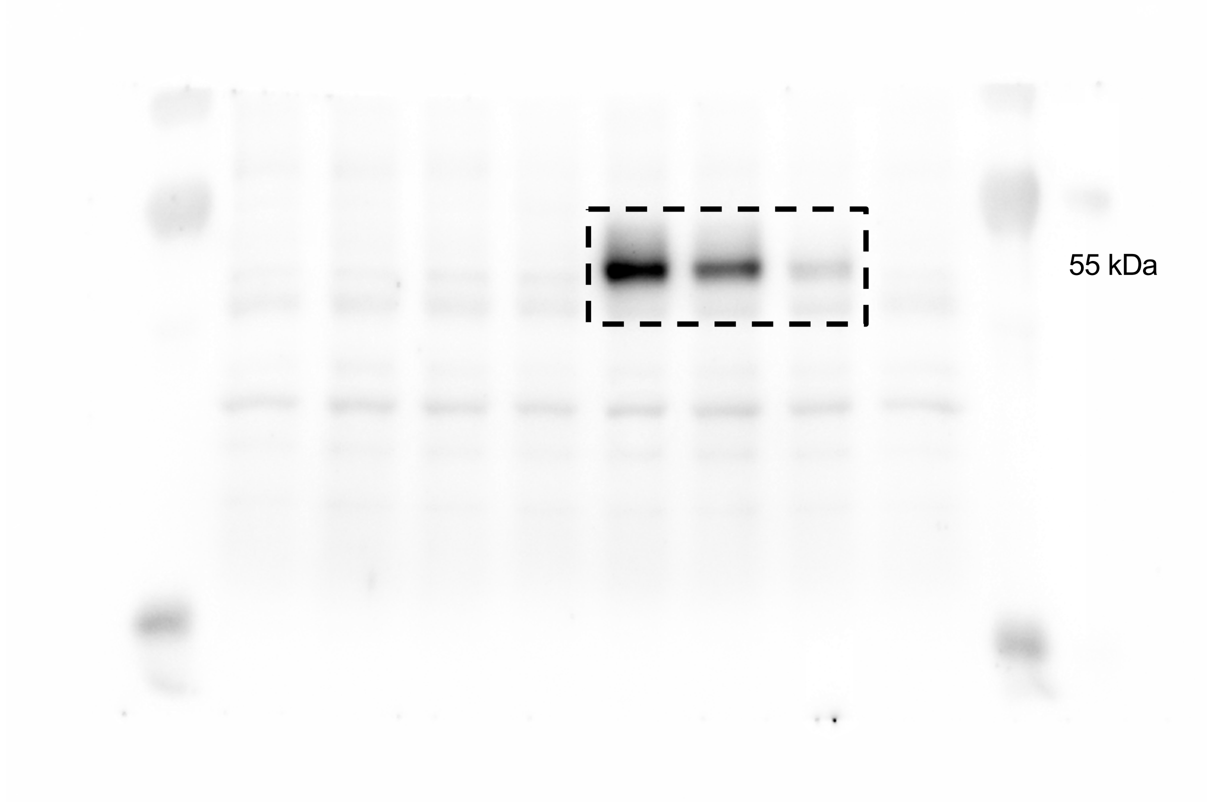

LC3-I, LC3-II

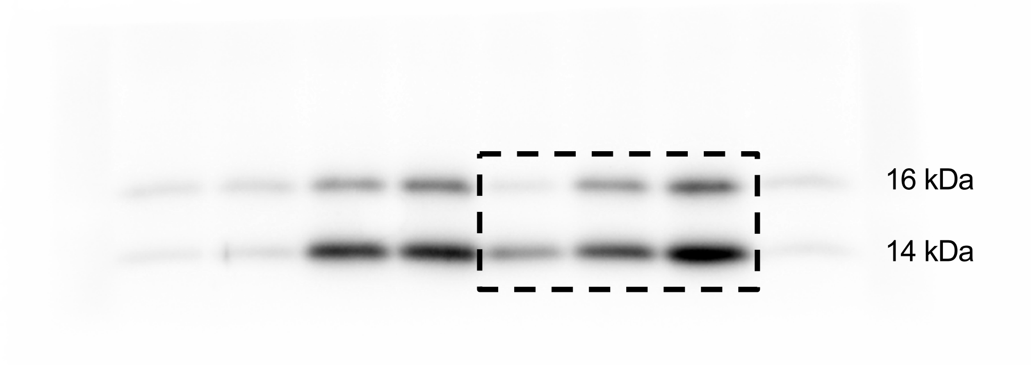

Beta actin

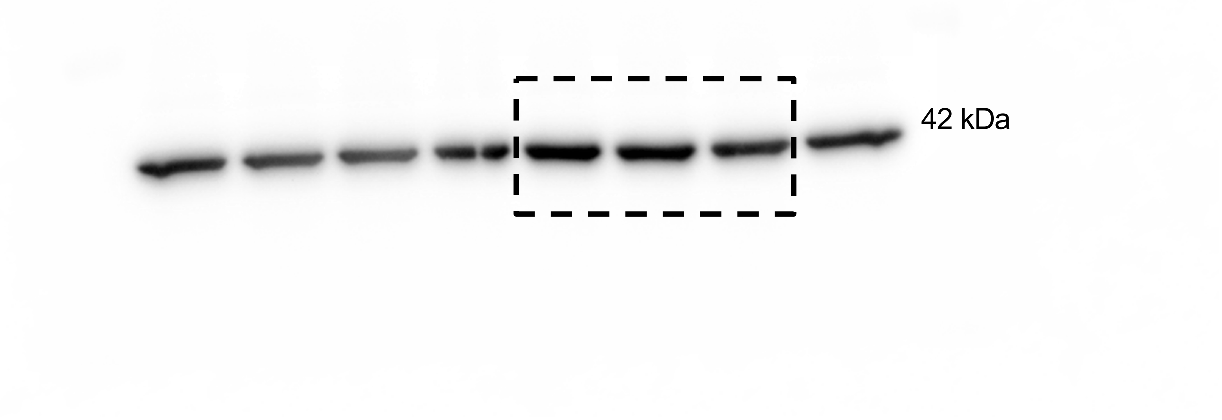

Fig. S9. Full-length images of the immunoblots in Fig. 3A. Black dot line boxes indicate the cropped images used in Fig. 3A.

Fig. S10

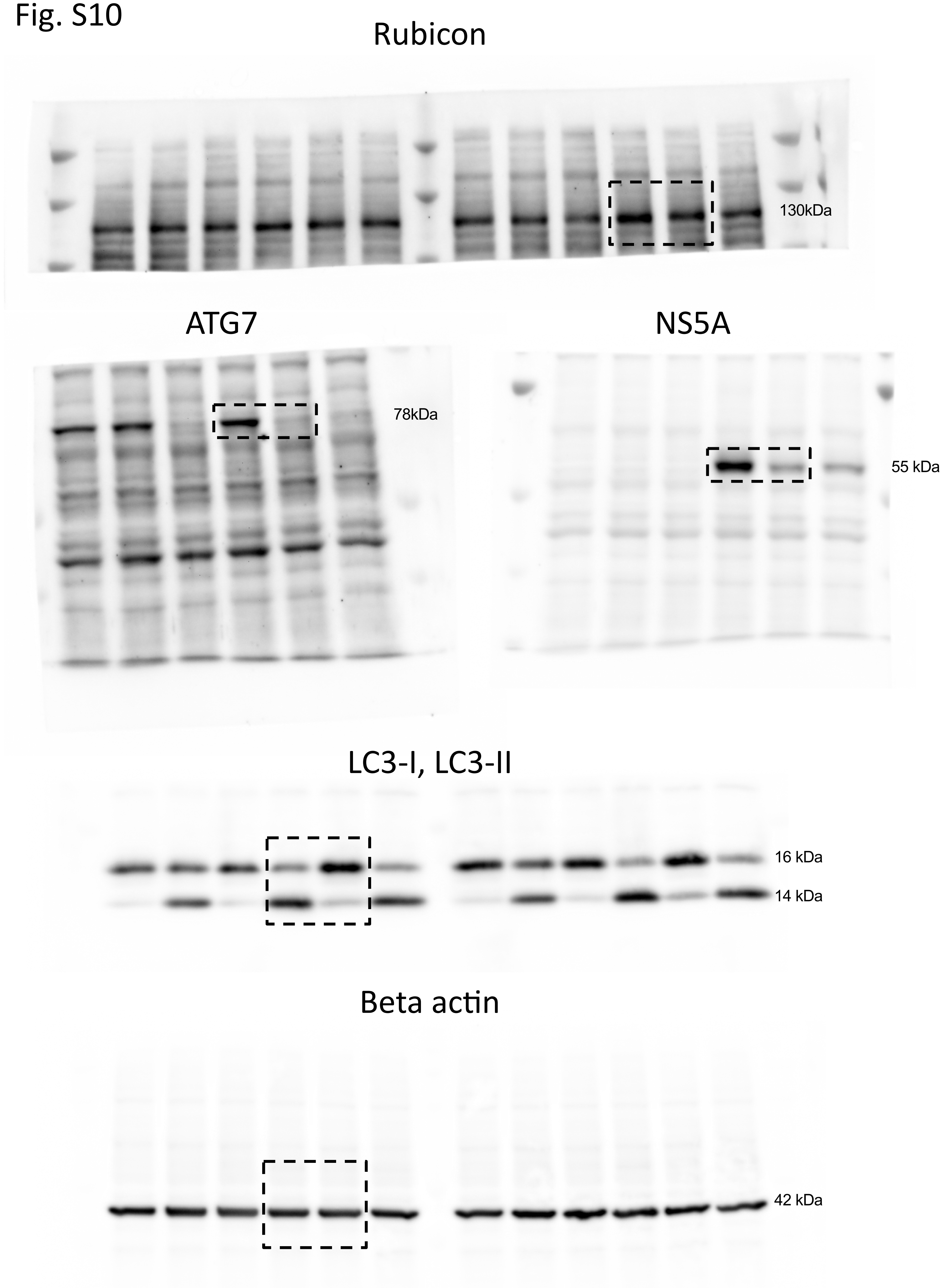

Fig. S10. Full-length images of the immunoblots in Fig. 3C. Black dot line boxes indicate the cropped images used in Fig. 3C.

Fig. S11

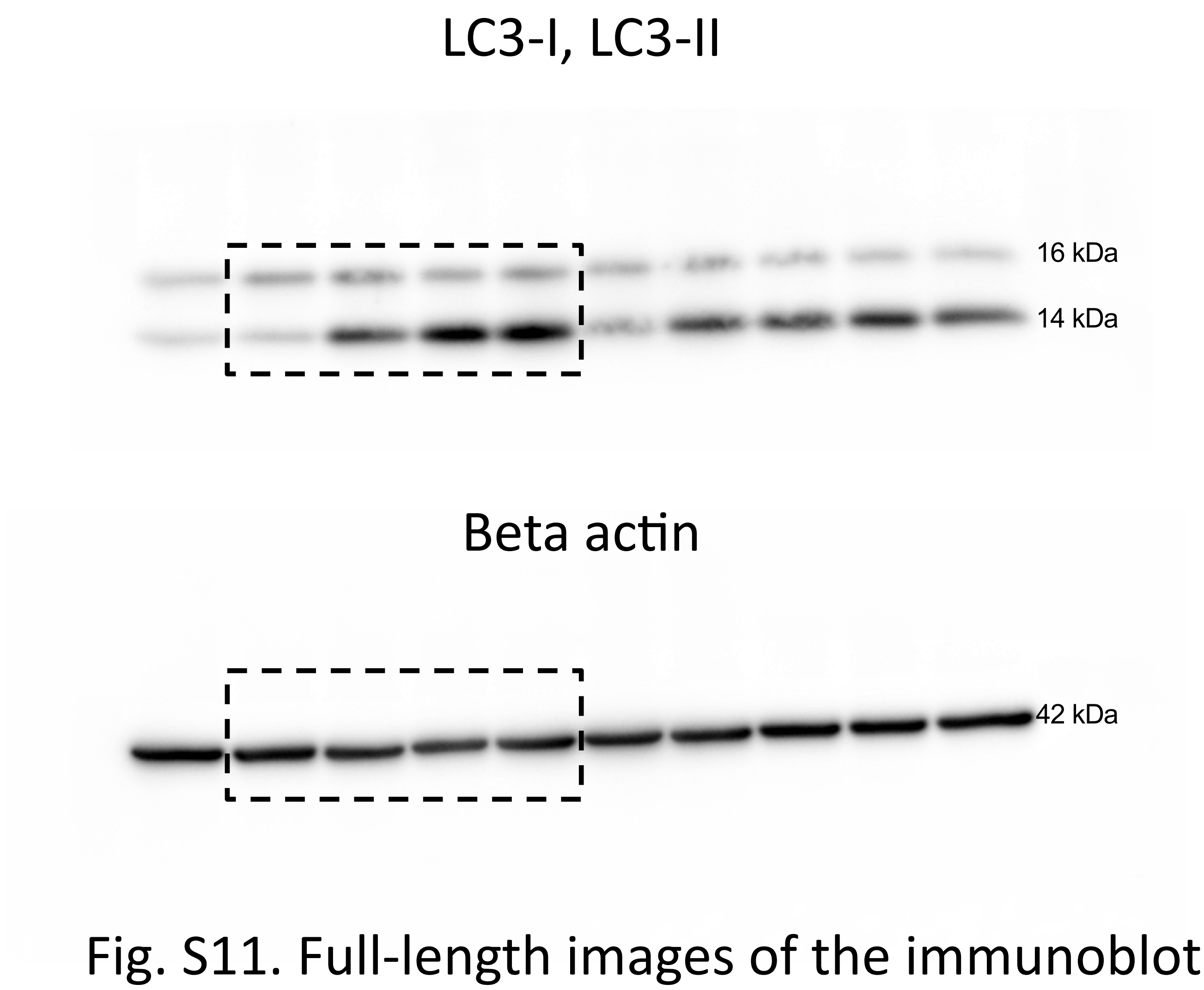

Fig. S11. Full-length images of the immunoblots in Sup Figure 1B. Black dot line boxes indicate the cropped images used in Sup Figure 1B.

Fig. S12

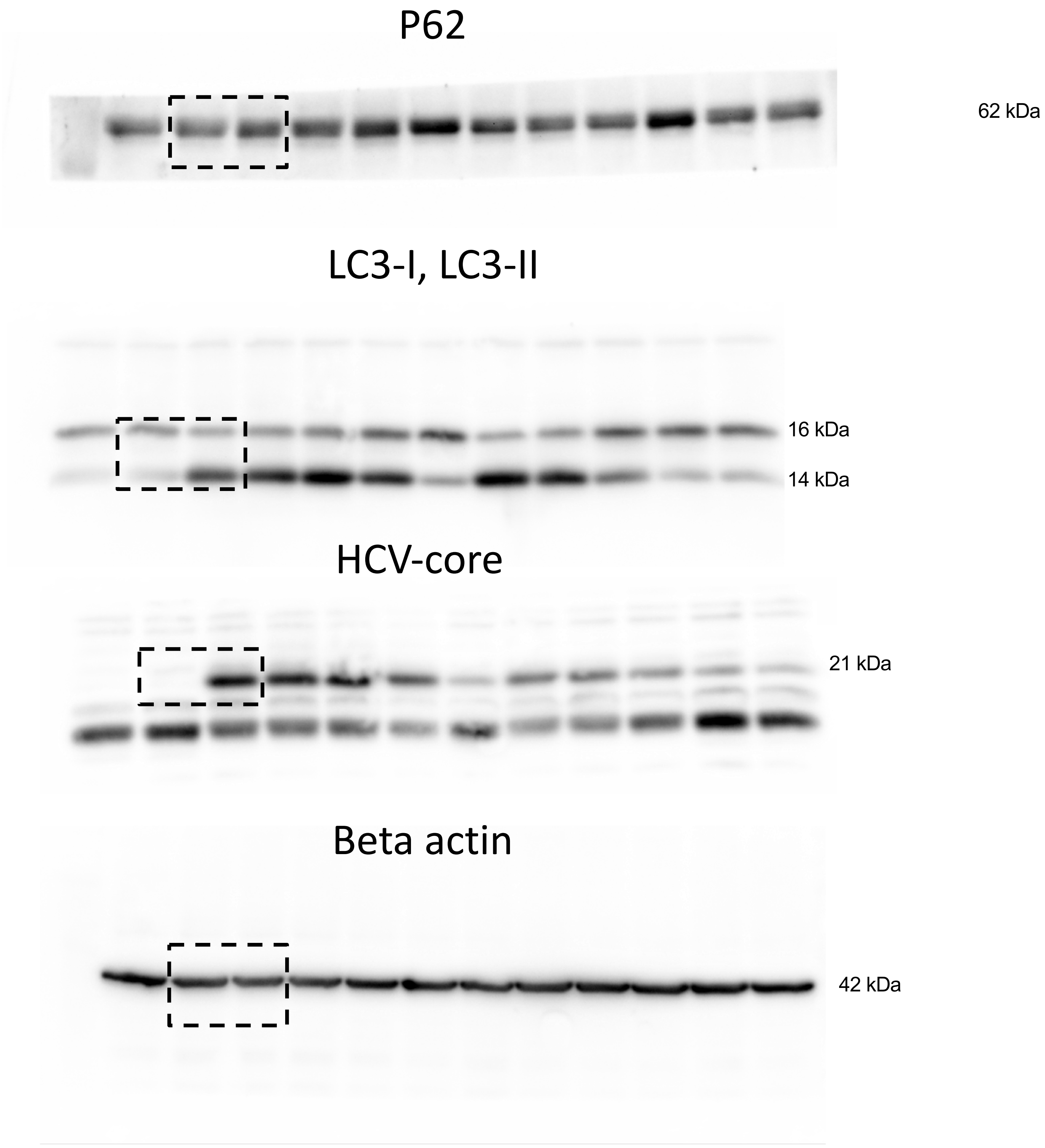

Fig. S11. Full-length images of the immunoblots in Sup Figure 1C. Black dot line boxes indicate the cropped images used in Sup Figure 1C.

Fig. S13

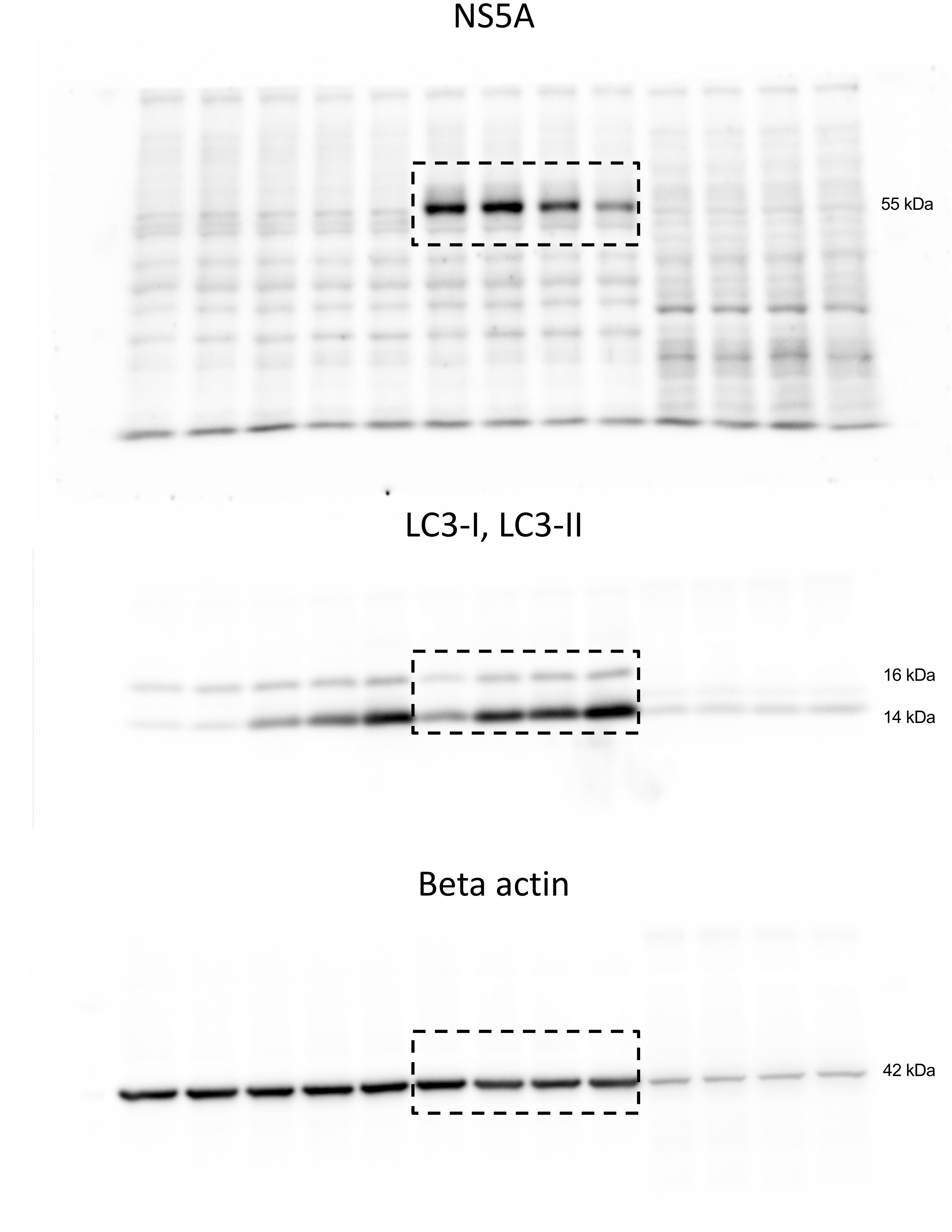

Fig. S13. Full-length images of the immunoblots in Sup Figure 3A. Black dot line boxes indicate the cropped images used in Sup Figure 3A.

Fig. S14

Rubicon

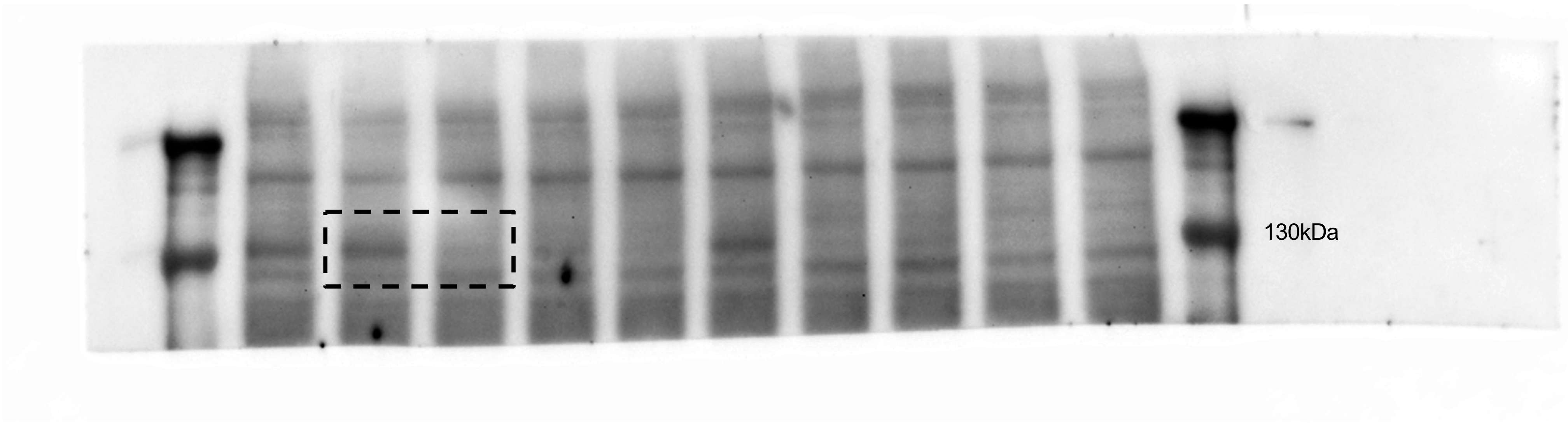

LC3-I, LC3-II

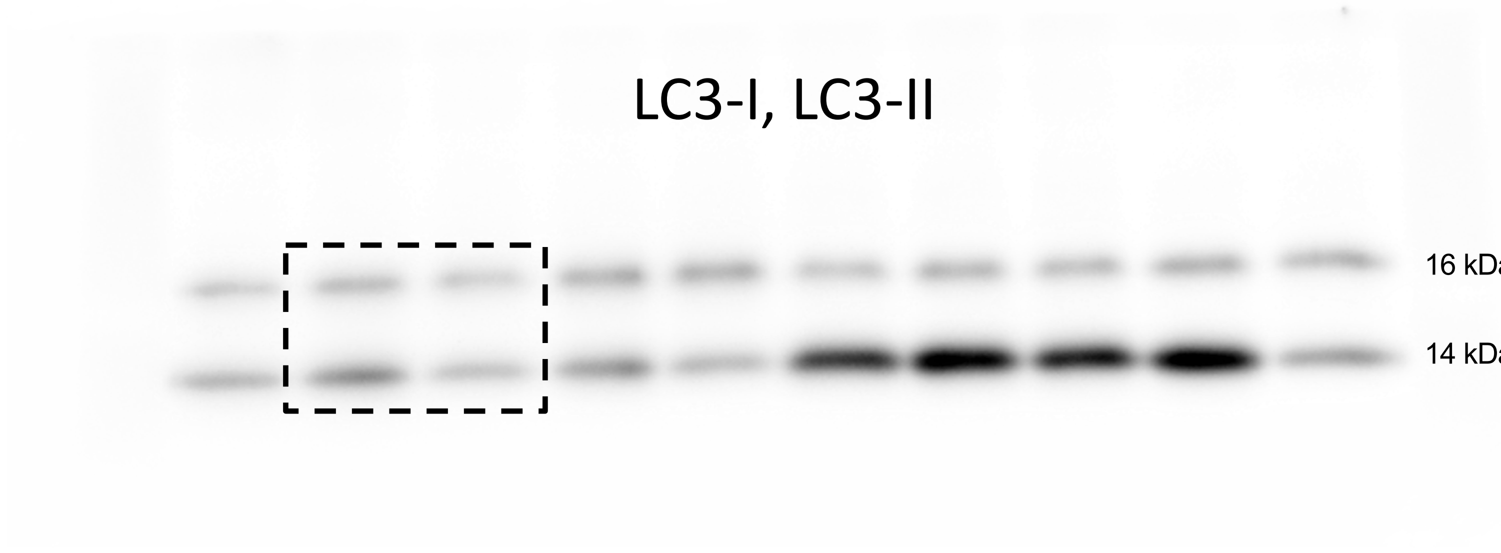

Beta actin

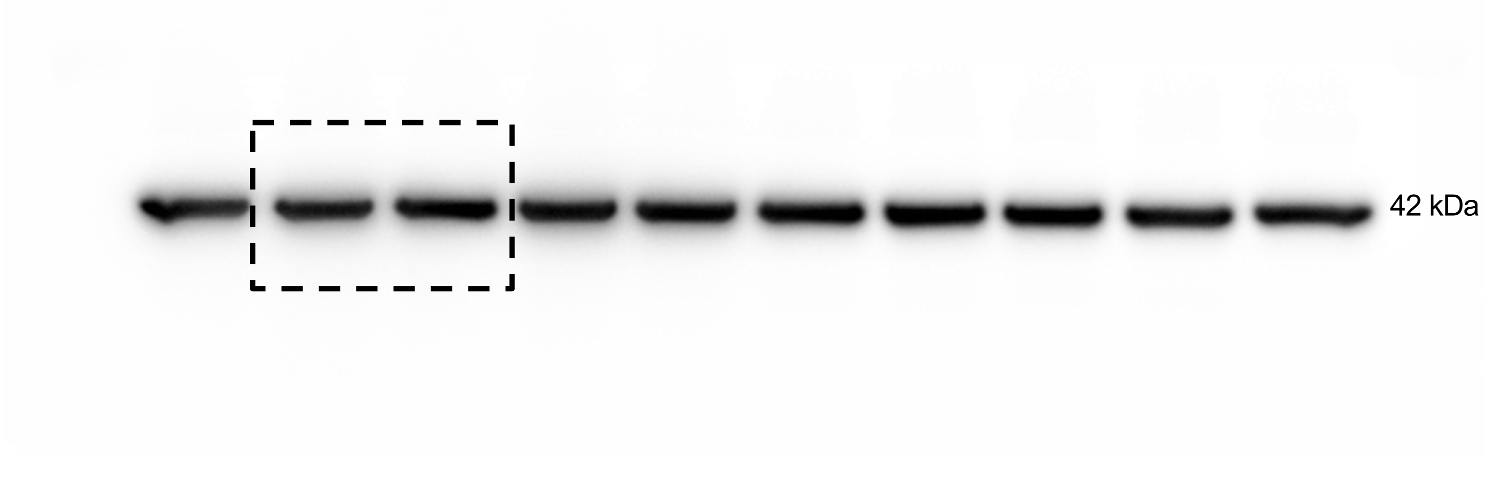

Fig. S14. Full-length images of the immunoblots in Sup Figure 4A. Black dot line boxes indicate the cropped images used in Sup Figure 4A.

Fig. S15

Rubicon

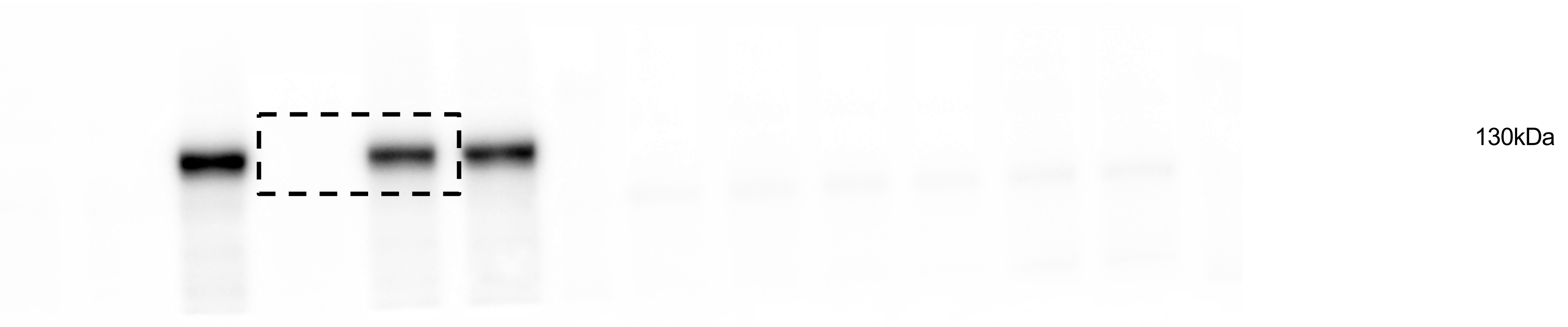

LC3-I, LC3-II

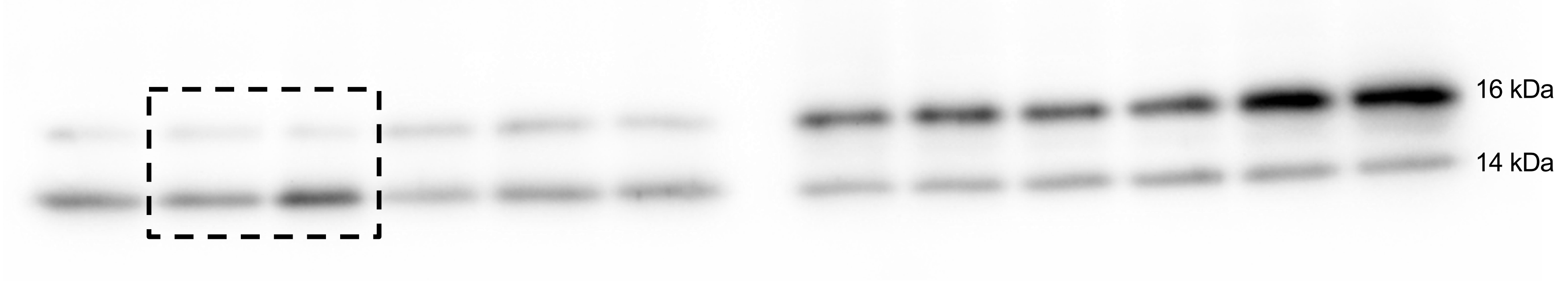

Beta actin

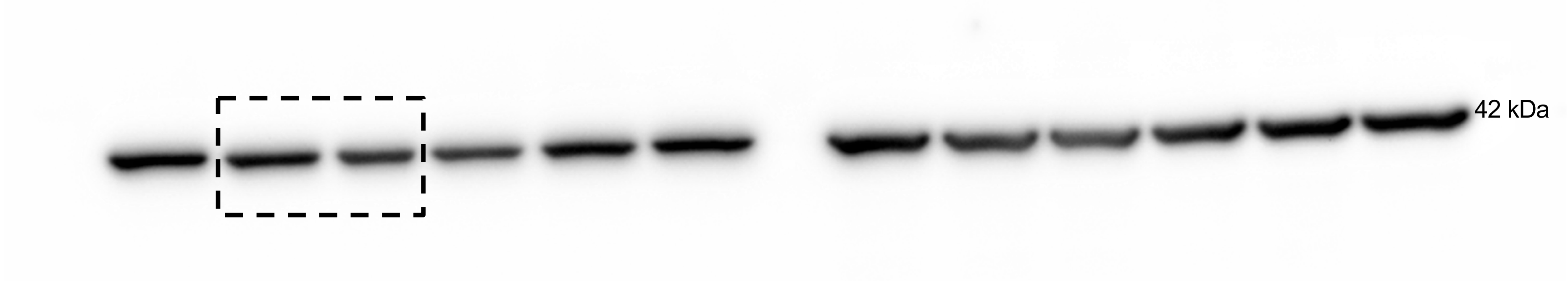

Fig. S15. Full-length images of the immunoblots in Sup Figure 4B. Black dot line boxes indicate the cropped images used in Sup Figure 4B.

Fig. S16

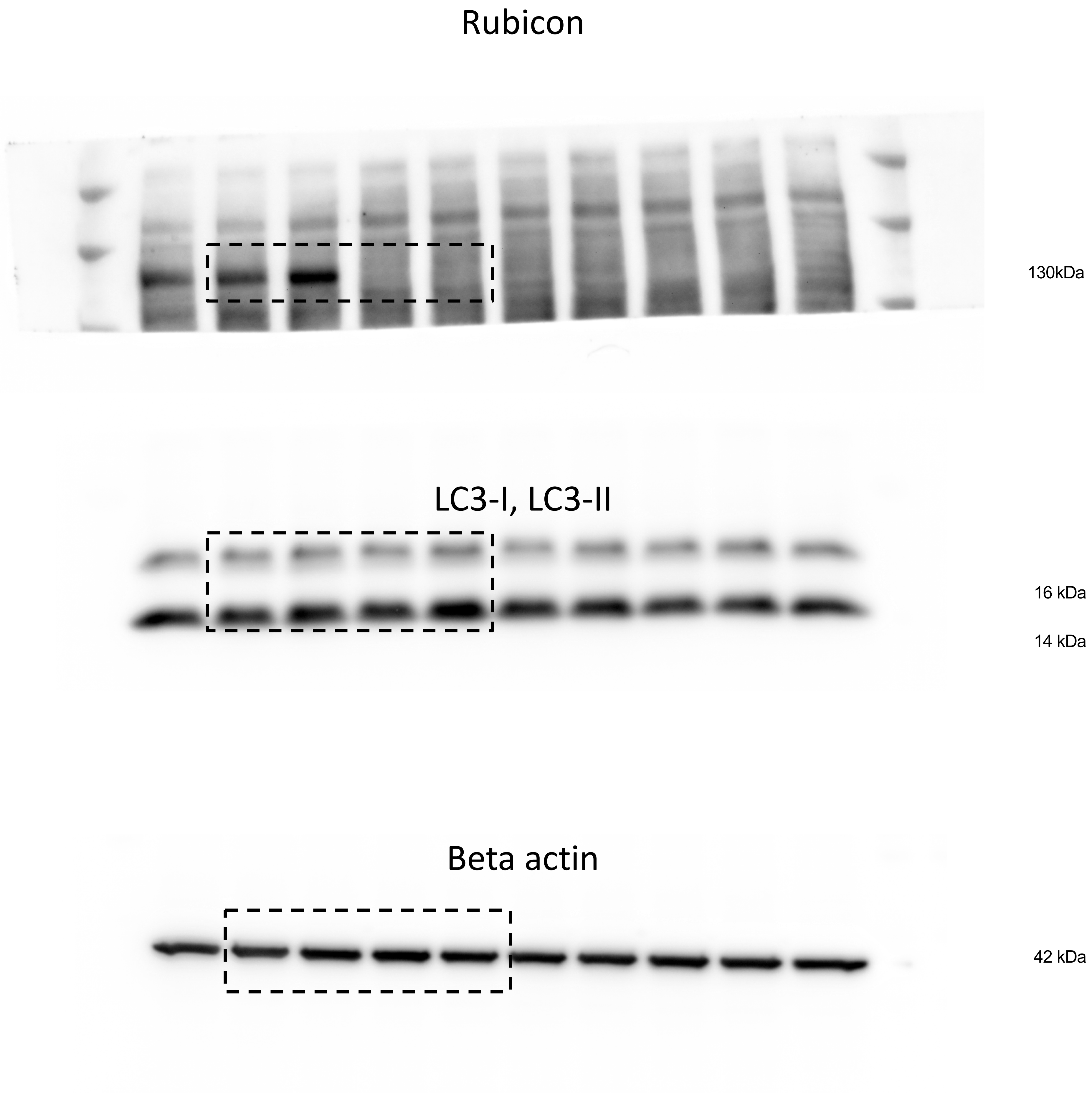

Fig. S16. Full-length images of the immunoblots in Sup Figure 4C. Black dot line boxes indicate the cropped images used in Sup Figure 4C.
