# Supplementary material for: Hepatitis C virus enhances Rubicon expression, leading to autophagy inhibition and intracellular innate immune activation
Source: Sci Rep. 2020 Sep 17;10:15290. doi: 10.1038/s41598-020-72294-y (PMC7498609; doi:10.1038/s41598-020-72294-y)
Supplement: Supplementary file 2 — Supplementary file2 [file 41598_2020_72294_MOESM2_ESM.docx]

**Title:**

**Hepatitis C virus enhances Rubicon expression, leading to autophagy inhibition and intracellular innate immune activation**

**Yuto Shiode^1^, Hayato Hikita^1^, Satoshi Tanaka^1^, Kumiko Shirai^1^, Akira Doi^1^, Sadatsugu Sakane^1^, Yugo Kai^1^, Tasuku Nakabori^1^, Ryoko Yamada^1^, Takahiro Kodama^1^, Ryohei Narumi^2^, Ryotaro Sakamori^1^, Hidetoshi Eguchi^3^, Takeshi Tomonaga^2^, Tomohide Tatsumi^1^ and Tetsuo Takehara^1*^**

**^1^ Department of Gastroenterology and Hepatology Osaka University Graduate School of Medicine, Suita 565-0871, Osaka, Japan.**

**^2^ Laboratory of Proteome Research/Proteome for Drug Discovery, National Institute of Biomedical Innovation, Health and Nutrition, 7-6-8, Saito-Asagi, Ibaraki, Osaka, 567-0085, Japan.**

**^3^ Department of Gastroenterological Surgery Osaka University Graduate School of Medicine, Suita 565-0871, Osaka, Japan.**

***This author is the corresponding author.**

**E-mail addresses**

**Yuto Shiode: yuyuyuto@gh.med.osaka-u.ac.jp**

**Hayato Hikita: hikita@gh.med.osaka-u.ac.jp**

**Satoshi Tanaka: st0305@gh.med.osaka-u.ac.jp**

**Kumiko Shirai:** [**kshirai0610@gh.med.osaka-u.ac.jp**](mailto:kshirai0610@gh.med.osaka-u.ac.jp)

**Akira Doi:** [**dokira0820@gh.med.osaka-u.ac.jp**](mailto:dokira0820@gh.med.osaka-u.ac.jp)

**Sadatsugu Sakane:** [**ssakane@gh.med.osaka-u.ac.jp**](mailto:ssakane@gh.med.osaka-u.ac.jp)

**Yugo Kai:** [**kaiyugo@gh.med.osaka-u.ac.jp**](mailto:kaiyugo@gh.med.osaka-u.ac.jp)

**Tasuku Nakabori:** [**t.nakabori@gh.med.osaka-u.ac.jp**](mailto:t.nakabori@gh.med.osaka-u.ac.jp)

**Ryoko Yamada:** [**ryo726@gh.med.osaka-u.ac.jp**](mailto:ryo726@gh.med.osaka-u.ac.jp)

**Takahiro Kodama:** [**t-kodama@gh.med.osaka-u.ac.jp**](mailto:t-kodama@gh.med.osaka-u.ac.jp)

**Ryohei Narumi:** [**narumi@nibiohn.go.jp**](mailto:narumi@nibiohn.go.jp)

**Ryotaro Sakamori:** [**sakamori@gh.med.osaka-u.ac.jp**](mailto:sakamori@gh.med.osaka-u.ac.jp)

**Hidetoshi Eguchi:** [**heguchi@gesurg.med.osaka-u.ac.jp**](mailto:heguchi@gesurg.med.osaka-u.ac.jp)

**Tsuyoshi Tomonaga: tomonaga@nibiohn.go.jp**

**Tomohide Tatsumi: tatsumit@gh.med.osaka-u.ac.jp**

**Tetsuo Takehara: takehara@gh.med.osaka-u.ac.jp**

**Subject terms: chronic hepatitis C, interferon-stimulated genes, direct-acting antivirals (DAAs)**

**Corresponding Author**

**Tetsuo Takehara, M.D., Ph.D.**

**2-2 Yamadaoka, Suita, Osaka, 565-0871 Japan**

**TEL: +81-6-6879-3621 FAX: +81-6-6879-3629**

**E-mail: takehara@gh.med.osaka-u.ac.jp**

**SUPPLEMENTAL FIGURE LEGENDS**

Sup. FIGURE 1

**A.** Representative confocal microscopy images of Huh7.5.1 cells infected with HCV (JFH-1) at a MOI of 3 for 3 days (upper panels) and Huh7 cells with or without HCV replicon transfection (lower panels). The cells were stained with DAPI (blue) and a monoclonal antibody against NS5A (green). **B.** LC3 turnover assay of Huh7 cells with or without HCV replicon transfection to determine autophagic flux (n = 3 each). **C.** Huh7.5.1 cells were infected with JFH-1 for 72 hours. Western blot analysis of P62, LC3 and HCV-core (left). The mRNA expression level of P62 (right). **D.** mRNA expression levels of Rubicon in Huh7 cells with or without HCV replicon transfection. **E.** Huh7.5.1 cells were infected with HCV (JFH-1) and then harvested at the indicated times. The mRNA expression level of HCV RNA. *; p<0.05.

Sup. FIGURE 2

HCV replicon-harbouring cells were transfected with siRNAs against Rubicon for 72 hours. Cells were also transfected with GFP-RUBICON or a negative control plasmid (GFP). The mRNA expression levels of RUBICON, IFNA1, IFNB1, ISG15, ISG56, and MX1 and HCV RNA levels. *; p<0.05

Sup. FIGURE 3

HCV replicon-harbouring cells were treated with chloroquine at the indicated concentrations for 48 hours. **A.** Western blot analysis of HCV NS5A and LC3. **B.** mRNA expression levels of type 1 interferon-related genes and HCV RNA levels in cells. **C.** HCV replicon-harbouring cells were transfected with three different siRNAs against Rubicon (siRub #1, #2 and #3) for 72 hours. The mRNA expression levels of ISG15, ISG56 and Mx1. **D.** HCV replicon-harbouring cells were transfected with GFP-ISG15, GFP-ISG56, GFP-Mx1 or a negative control plasmid (GFP). The mRNA expression levels of ISG15, ISG56, and Mx1 and HCV RNA levels. *; p<0.05

Sup. FIGURE 4

**A.** Huh 7 cells were transfected with a siRNA against Rubicon for 72 hours. Western blot analysis of Rubicon and LC3. **B.** Huh 7 cells were transfected with GFP-RUBICON or a negative control plasmid (GFP). Western blot analysis of Rubicon and LC3. **C.** LC3 turnover assay of HCV replicon-harbouring cells transfected with a siRNA against Rubicon for 72 hours (n = 3 each). *; p<0.05
