# Supplementary figures and images for: Hepatitis C virus enhances Rubicon expression, leading to autophagy inhibition and intracellular innate immune activation
Source: Sci Rep. 2020 Sep 17;10:15290. doi: 10.1038/s41598-020-72294-y (PMC7498609; doi:10.1038/s41598-020-72294-y)

A

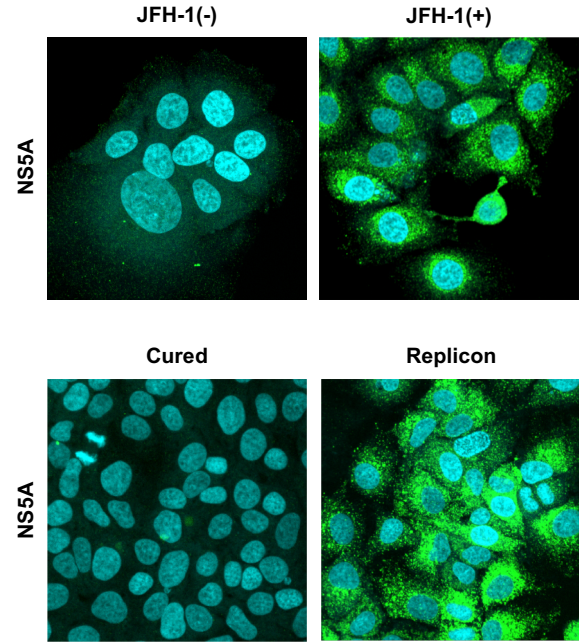

B

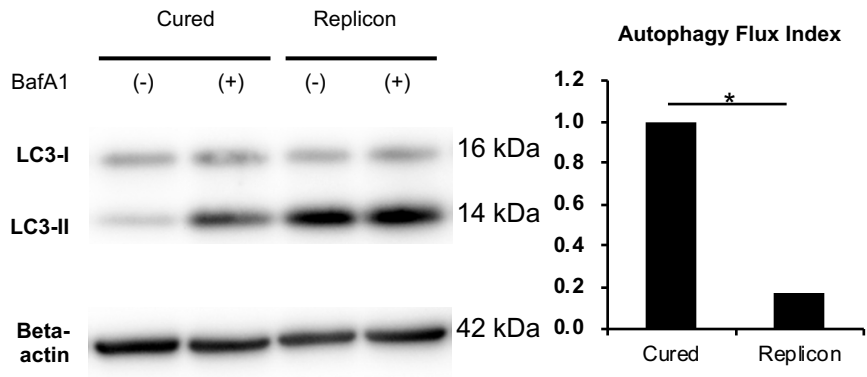

C

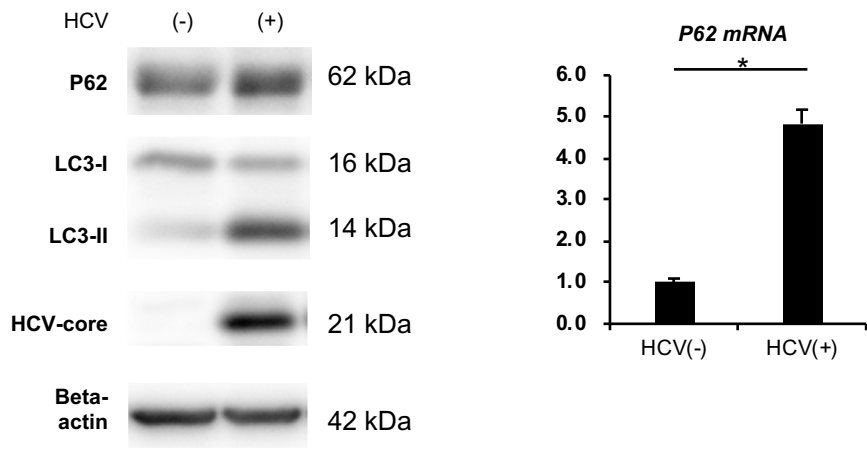

D

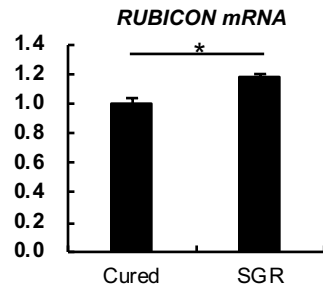

E

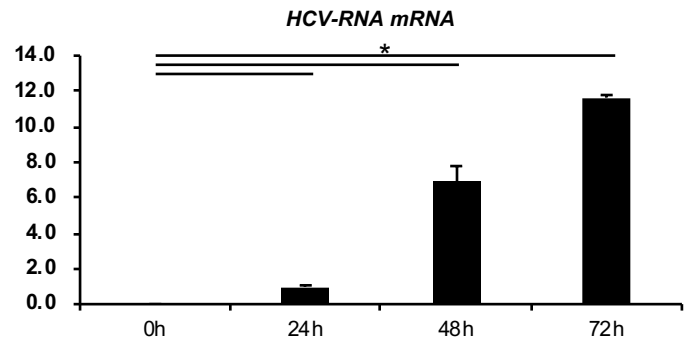

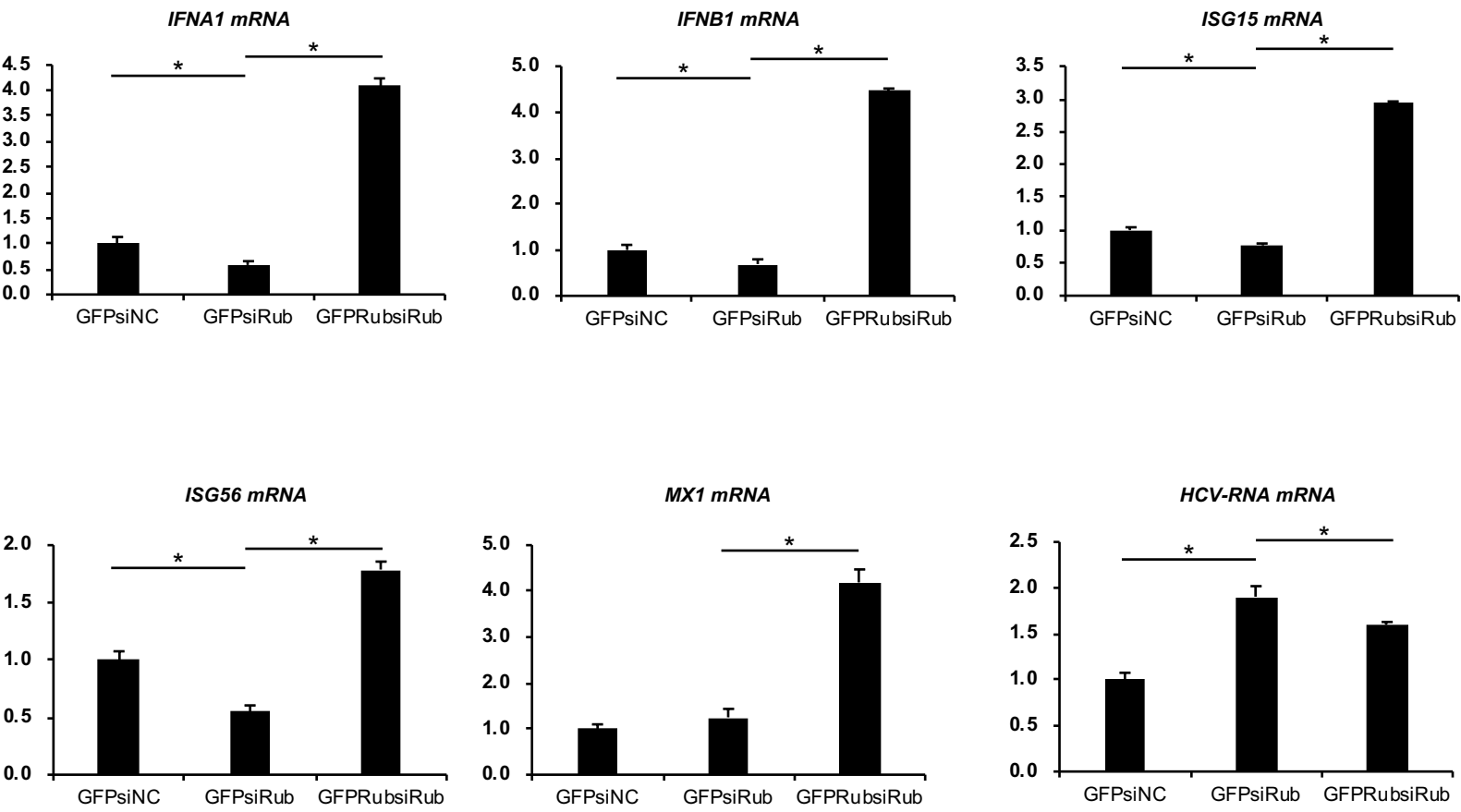

A

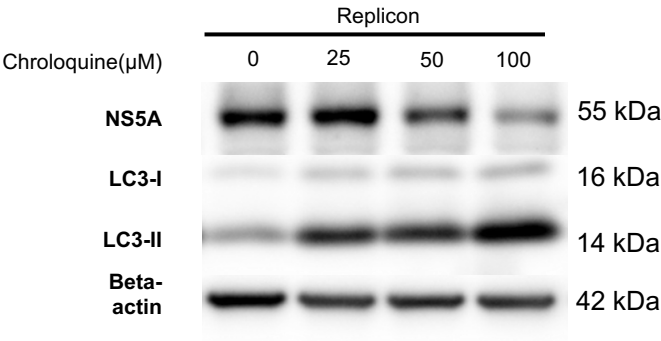

B

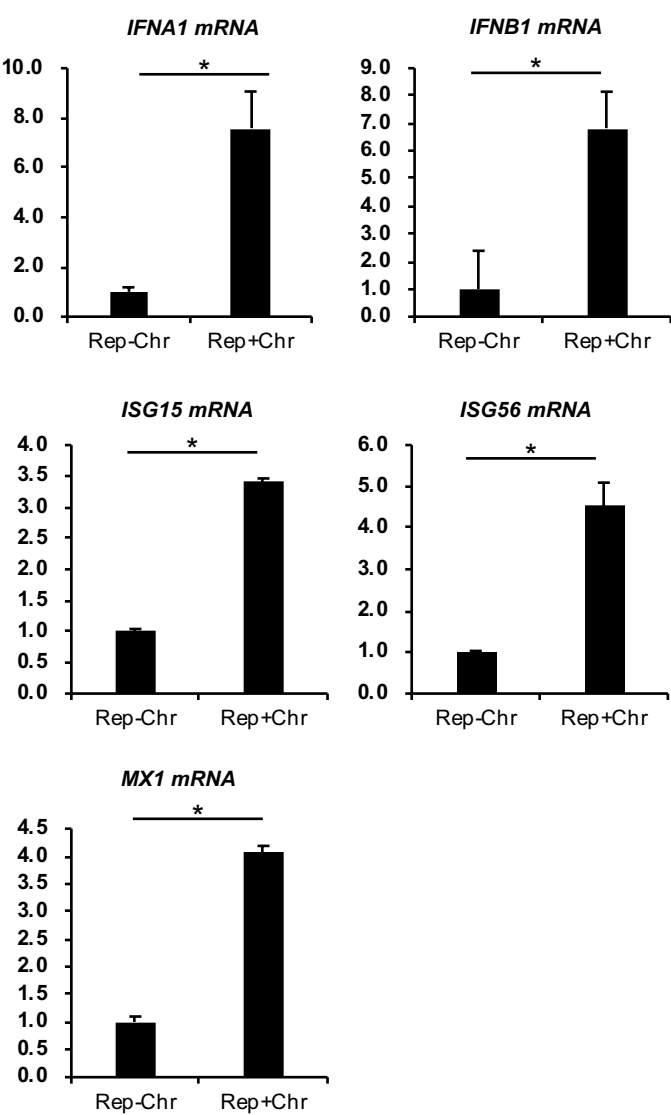

C

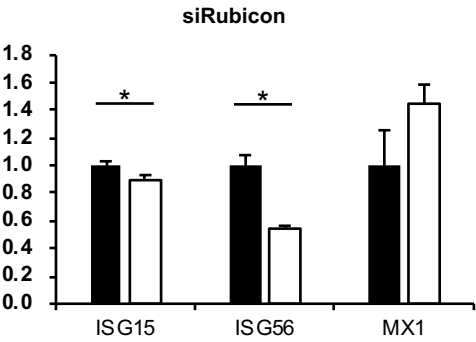

D

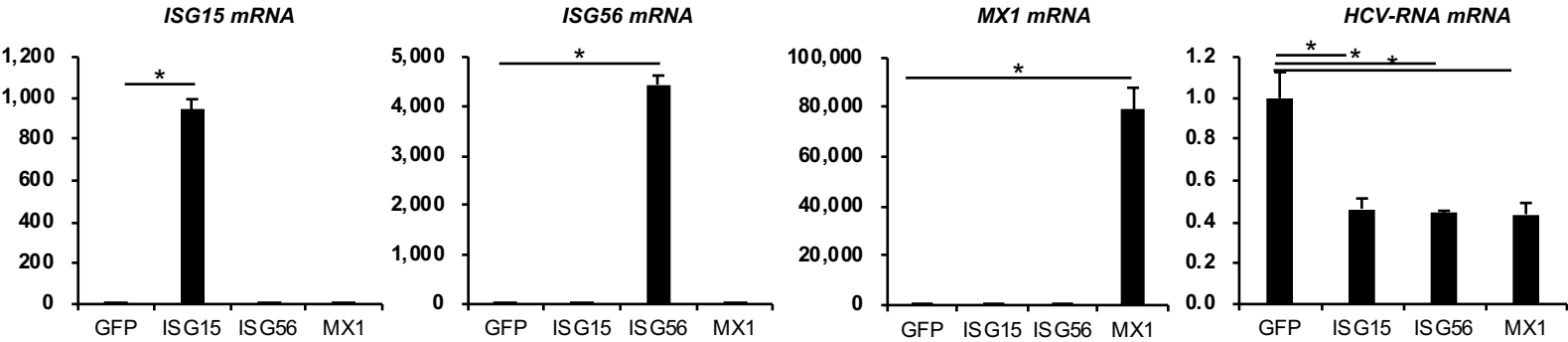

A

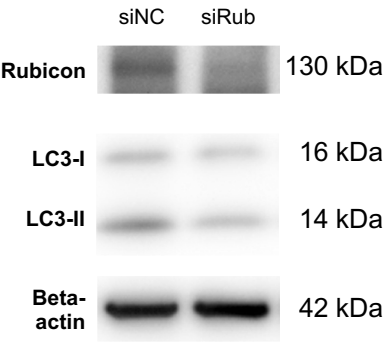

B

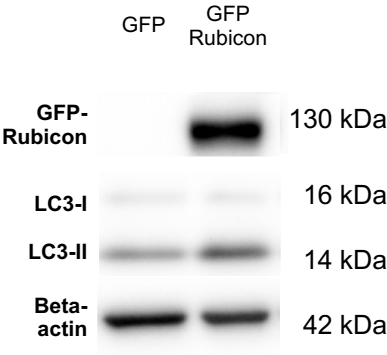

C

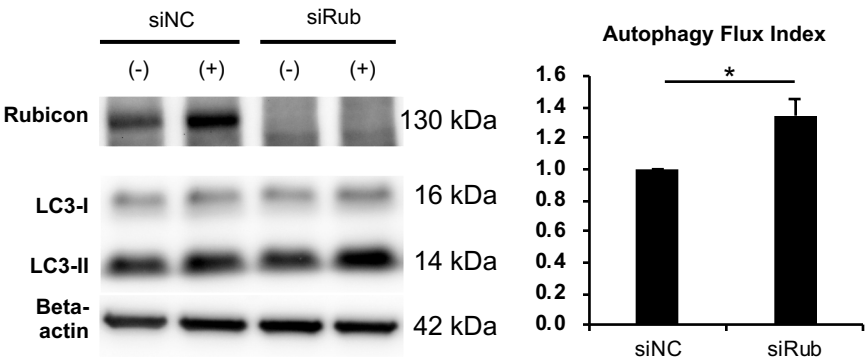

Supplement: Supplementary file 3 — Supplementary file3 [file 41598_2020_72294_MOESM3_ESM.pdf]
