# Supplementary material for: Hepatitis C virus enhances Rubicon expression, leading to autophagy inhibition and intracellular innate immune activation
Source: Sci Rep. 2020 Sep 17;10:15290. doi: 10.1038/s41598-020-72294-y (PMC7498609; doi:10.1038/s41598-020-72294-y)
Supplement: Supplementary file 4 — Supplementary file4 [file 41598_2020_72294_MOESM4_ESM.docx]

Sup. Table 1

List of proteins whose differential expression analysis yielded a p value less than 0.05 by proteome analysis using the livers of HCV-infected mice (n = 3) and those of uninfected mice (n = 3).

| Protein name | Gene Name | Fold Change | t-test < |
| --- | --- | --- | --- |
| YTDC2_HUMAN Probable ATP-dependent RNA helicase YTHDC2 | YTHDC2 | 0.538924358 | 0.046775 |
| PCY1A_HUMAN Choline-phosphate cytidylyltransferase A | PCYT1A | 0.569399182 | 0.012524 |
| MD1L1_HUMAN Mitotic spindle assembly checkpoint protein MAD1 | MAD1L1 | 0.658949952 | 0.029834 |
| PTBP3_HUMAN Polypyrimidine tract-binding protein 3 | PTBP3 | 0.66943322 | 0.041884 |
| IRGQ_HUMAN Immunity-related GTPase family Q protein | IRGQ | 0.780153211 | 0.039271 |
| PGP_HUMAN Phosphoglycolate phosphatase | PGP | 0.8188842 | 0.022535 |
| PURB_HUMAN Transcriptional activator protein Pur-beta | PURB | 0.887423759 | 0.00676 |
| ARP5L_HUMAN Actin-related protein 2/3 complex subunit 5-like protein | ARPC5L | 0.89011164 | 0.039796 |
| SPCS_HUMAN O-phosphoseryl-tRNA(Sec) selenium transferase | SEPSECS | 0.909305668 | 0.018065 |
| DSRAD_HUMAN Double-stranded RNA-specific adenosine deaminase | ADAR | 1.076530141 | 0.037776 |
| NCBP1_HUMAN Nuclear cap-binding protein subunit 1 | NCBP1 | 1.089293587 | 0.027445 |
| CRTAP_HUMAN Cartilage-associated protein | CRTAP | 1.093709981 | 0.007104 |
| RT26_HUMAN 28S ribosomal protein S26, mitochondrial | MRPS26 | 1.132412935 | 0.037207 |
| GPX2_HUMAN Glutathione peroxidase 2 | GPX2 | 1.155521181 | 0.014435 |
| PSB8_HUMAN Proteasome subunit beta type-8 | PSMB8 | 1.158562146 | 0.035279 |
| SPCS1_HUMAN Signal peptidase complex subunit 1 | SPCS1 | 1.164960066 | 0.022673 |
| SIAT1_HUMAN Beta-galactoside alpha-2,6-sialyltransferase 1 | ST6GAL1 | 1.166092941 | 0.046455 |
| PLCE_HUMAN 1-acyl-sn-glycerol-3-phosphate acyltransferase epsilon | AGPAT5 | 1.17059065 | 0.024037 |
| DTX3L_HUMAN E3 ubiquitin-protein ligase DTX3L | DTX3L | 1.172264356 | 0.044355 |
| GBP1_HUMAN Interferon-induced guanylate-binding protein 1 | GBP1 | 1.190356512 | 0.031945 |
| GHC1_HUMAN Mitochondrial glutamate carrier 1 | SLC25A22 | 1.193582215 | 0.038179 |
| FRK_HUMAN Tyrosine-protein kinase FRK | FRK | 1.205847891 | 0.00565 |
| CREL1_HUMAN Cysteine-rich with EGF-like domain protein 1 | CRELD1 | 1.22682765 | 0.045573 |
| TPSN_HUMAN Tapasin | TAPBP | 1.230520878 | 0.00643 |
| S38A4_HUMAN Sodium-coupled neutral amino acid transporter 4 | SLC38A4 | 1.253526387 | 0.040791 |
| UB2L6_HUMAN Ubiquitin/ISG15-conjugating enzyme E2 L6 | UBE2L6 | 1.260112762 | 0.016613 |
| STAT1_HUMAN Signal transducer and activator of transcription 1-alpha/beta | STAT1 | 1.281509255 | 0.002711 |
| PIGR_HUMAN Polymeric immunoglobulin receptor | PIGR | 1.289897406 | 0.012049 |
| SAMH1_HUMAN Deoxynucleoside triphosphate triphosphohydrolase SAMHD1 | SAMHD1 | 1.294192526 | 0.039125 |
| BT3A3_HUMAN Butyrophilin subfamily 3 member A3 | BTN3A3 | 1.347143508 | 0.012973 |
| PNKD_HUMAN Probable hydrolase PNKD | PNKD | 1.366522139 | 0.046424 |
| PSB9_HUMAN Proteasome subunit beta type-9 | PSMB9 | 1.380129487 | 0.003607 |
| NFKB2_HUMAN Nuclear factor NF-kappa-B p100 subunit | NFKB2 | 1.382806328 | 0.012266 |
| OAS3_HUMAN 2'-5'-oligoadenylate synthase 3 | OAS3 | 1.393798539 | 0.005754 |
| IFIT2_HUMAN Interferon-induced protein with tetratricopeptide repeats 2 | IFIT2 | 1.409113937 | 0.034583 |
| DCK_HUMAN Deoxycytidine kinase | DCK | 1.444595022 | 0.000995 |
| IFIT1_HUMAN Interferon-induced protein with tetratricopeptide repeats 1 | IFIT1 | 1.497466475 | 0.000549 |
| ELMO2_HUMAN Engulfment and cell motility protein 2 | ELMO2 | 1.575558071 | 0.024465 |
| DDX60_HUMAN Probable ATP-dependent RNA helicase DDX60 | DDX60 | 1.672440826 | 0.001355 |
| 1B13_HUMAN HLA class I histocompatibility antigen, B-13 alpha chain;1B73_HUMAN HLA class I histocompatibility antigen, B-73 alpha chain | HLA-B;HLA-B | 1.699682793 | 0.04164 |
| 1A11_HUMAN HLA class I histocompatibility antigen, A-11 alpha chain | HLA-A | 1.824879993 | 0.00667 |
| ICAM1_HUMAN Intercellular adhesion molecule 1 | ICAM1 | 1.967915903 | 0.0005 |
| 1C07_HUMAN HLA class I histocompatibility antigen, Cw-7 alpha chain | HLA-C | 1.982305916 | 0.015964 |
| OAS2_HUMAN 2'-5'-oligoadenylate synthase 2 | OAS2 | 2.296706863 | 0.000894 |
| ISG15_HUMAN Ubiquitin-like protein ISG15 | ISG15 | 2.357213422 | 0.001202 |
